# Extension of Lifespan in *C. elegans* by Naphthoquinones That Act through Stress Hormesis Mechanisms

**DOI:** 10.1371/journal.pone.0021922

**Published:** 2011-07-13

**Authors:** Piper R. Hunt, Tae Gen Son, Mark A. Wilson, Quian-Sheng Yu, William H. Wood, Yongqing Zhang, Kevin G. Becker, Nigel H. Greig, Mark P. Mattson, Simonetta Camandola, Catherine A. Wolkow

**Affiliations:** 1 Laboratory of Neurosciences, National Institute on Aging (NIA) Intramural Research Program, National Institutes of Health (NIH), Baltimore, Maryland, United States of America; 2 Drug Design and Development Section, National Institute on Aging (NIA) Intramural Research Program, National Institutes of Health (NIH), Baltimore, Maryland, United States of America; 3 Gene Expression and Genomics Unit, National Institute on Aging (NIA) Intramural Research Program, National Institutes of Health (NIH), Baltimore, Maryland, United States of America; University of Dundee, United Kingdom

## Abstract

Hormesis occurs when a low level stress elicits adaptive beneficial responses that protect against subsequent exposure to severe stress. Recent findings suggest that mild oxidative and thermal stress can extend lifespan by hormetic mechanisms. Here we show that the botanical pesticide plumbagin, while toxic to *C. elegans* nematodes at high doses, extends lifespan at low doses. Because plumbagin is a naphthoquinone that can generate free radicals in vivo, we investigated whether it extends lifespan by activating an adaptive cellular stress response pathway. The *C. elegans* cap‘n’collar (CNC) transcription factor, SKN-1, mediates protective responses to oxidative stress. Genetic analysis showed that *skn-1* activity is required for lifespan extension by low-dose plumbagin in *C. elegans*. Further screening of a series of plumbagin analogs identified three additional naphthoquinones that could induce SKN-1 targets in *C. elegans*. Naphthazarin showed *skn-1*dependent lifespan extension, over an extended dose range compared to plumbagin, while the other naphthoquinones, oxoline and menadione, had differing effects on *C. elegans* survival and failed to activate ARE reporter expression in cultured mammalian cells. Our findings reveal the potential for low doses of naturally occurring naphthoquinones to extend lifespan by engaging a specific adaptive cellular stress response pathway.

## Introduction

As organisms age, cellular proteins, lipids and nucleic acids sustain damage that can lead to functional deficits in tissues and, ultimately, death. The free radical theory of aging proposes that aging results, at least in part, from damage to cellular components by reactive oxygen species (ROS), such as nitroxides, hydrogen peroxide and superoxide anion. Indeed, oxidative modification is a major form of damage detected in aging tissues [1]–[3]. ROS occur as byproducts of normal mitochondrial metabolism, but are also produced by environmental sources, including some biological toxins. Levels of oxidative damage correlate with relative age and extent of functional decline, consistent with oxidative damage acting as a contributing force driving tissue decline with age [4], [5]. Aging-related diseases, such as Alzheimer's disease and cancer, have also been linked to oxidative damage [6], [7]. Multiple lines of evidence suggest that attenuating stressful insults or increasing stress resistance can delay aging and functional decline in model organisms and in human tissues [8]–[10]. Such data support the concept that chemicals with prolongevity activity can be identified by their ability to activate stress response pathways.

Stress hormesis occurs when toxic agents elicit beneficial effects at low concentrations and is classically described by an inverted U-shaped dose response curve [11]. Stress hormesis has been observed for both thermal and oxidative stressors. Sublethal thermal stress is protective against subsequent thermal stress in yeast, worms, and flies [12]–[14]. In *Caenorhabditis elegans*, sublethal thermal stress can extend adult lifespan, suggesting that thermal stress responses overlap with prolongevity pathways in this organism [13], [15], [16]. Chemical stress can induce heat shock protein expression and thermotolerance in *Saccharomyces cerevisiae* [17]. Cultured cells and whole organisms are protected from oxidative stress by pretreatments with hyperbaric oxygen or low levels of free-radical generators such as paraquat or juglone [16], [18]. In addition, mild oxidative stress from low concentrations of juglone extended *C. elegans* lifespan, suggesting that oxidative stress response pathways also overlap with prolongevity pathways in *C. elegans* [19]. The close link between stress and aging suggests that interventions harnessing hormetic mechanisms may extend lifespan or delay age-associated functional decline. However, challenges for developing hormetic mechanisms into anti-aging therapies include the relatively small dose range providing protective benefits and the toxic effects of higher doses. Therefore, studies are needed to determine the feasibility of modifying hormetic agents to extend the beneficial dose range and minimize toxicity.

Here, we report that hormetic chemicals can be modified to optimize beneficial effects and minimize toxicity in *C. elegans*, a model for studying aging in whole organisms. *C. elegans* is well-suited to this problem due to the short lifespan, ease of genetic manipulation and transparent anatomy. First, we examined whether lifespan extension is common among biological toxins with various chemical structures and mechanisms of action. In a small screen of natural phytochemicals, we identified two ROS generating compounds, plumbagin and juglone, which extended lifespan at subtoxic doses. Mean lifespan extension by plumbagin was dependent on SKN-1, a cap‘n’collar transcription factor that promotes antioxidant gene expression in response to oxidative stress [20]. We further screened a collection of six plumbagin analogs, identifying three additional naphthoquinones that activated expression of a *skn-1* target. One of these could extend lifespan over a larger range of doses than plumbagin, demonstrating the utility of stress hormesis mechanisms as promising prolongevity intervention. The other compounds had differing effects on longevity, possibly reflecting structure-specific alterations in stability and toxicity. This work highlights *C. elegans* as an experimental approach for identifying lead compounds with the potential to act on conserved targets.

## Results

### A screen for biological toxins with hormetic prolongevity activity

To gain a better understanding about the ability of phytotoxins to extend lifespan through stress hormesis mechanisms, we conducted a small screen of 14 phytochemicals which was derived from a larger collection of 30 phytochemicals used to identify compounds activating stress response pathways in cultured cells [21]. These compounds represented a diverse range of chemicals, including lignins, lipids, alcohols and cyclic compounds, encompassing at least three modes of action, ROS generators, antifeedants and neurotoxins (Table 1). For the *C. elegans* screen, we examined survival of populations of roughly 40 sterile *fem-1(hc17)* adults treated with each of the 14 phytochemicals [22]. First, each phytotoxin was tested for toxicity at 200 µM. Four phytotoxins were toxic at this dose (plumbagin, visnagin, eugenol and farnesol) and were retested at 100 µM (Table 1). At 100 µM, toxicity was observed for plumbagin, visnagin and eugenol, while farnesol had no effect. Plumbagin, visnagin and eugenol were further tested at 60, 30 and 10 µM doses. At both 60 and 30 µM, plumbagin demonstrated a prolongevity effect with treated animals surviving longer than controls. A previous study reported that 50 µM plumbagin was toxic to *C. elegans* [23]. This variation in hormetic and toxic dose ranges for plumbagin may reflect variability in culturing conditions between laboratories. We further tested 300 µM doses for 10 phytotoxins that were not toxic at 200 µM. Of these, juglone, piperine, veratrine and asarone were toxic at 300 µM, although they had shown no benefit or toxicity at 200 µM. We examined these at 100 µM and found that juglone was beneficial and could extend lifespan at this concentration. In an independent study, 40 µM juglone extended *C. elegans* lifespan in axenic media [19].

**Table 1:** Screen for hormetic prolongevity activity in *C. elegans*.

Mechanism	Compound	Notes	Relative survival; (µM dose)					
			300	200	100	60	30	10
ROS generators	Plumbagin	antiproliferative yellow pigment		<	<	>	>	≈
	Juglone	herbicidal brown pigment	<	≈	>			
	Visnagin	vasodilator		<	<	<	<	≈
Neurotoxins	Eugenol	TRPV channel agonist		<	<	≈	≈	≈
	Piperine	TRPV channel agonist	<	≈	≈			
	Veratrine	Na+ channel antagonist	<	≈	≈			
	Anabasine	Nicotinic agonist	≈	≈	≈			
	Domoic acid	Neurotoxin	≈	≈				
Antifeedants	Farnesol	Membrane disruptor Sesquiterpenoid		<	≈			
	a-Asarone	Phenylpropanoid	<	≈	≈			
	Marmesin	Coumarin	≈	≈				
	Precocene II	Cytotoxin	≈	≈				
	Sesamin	Lignin	≈	≈				
Translation inhibitor	L-canavanine	Arginine analog	≈	≈				

Empty cells, dose not tested; (<) shorter survival than untreated controls; (≈) approximately equal survival to untreated controls; (>) longer survival than untreated controls.

**Figure 1:**
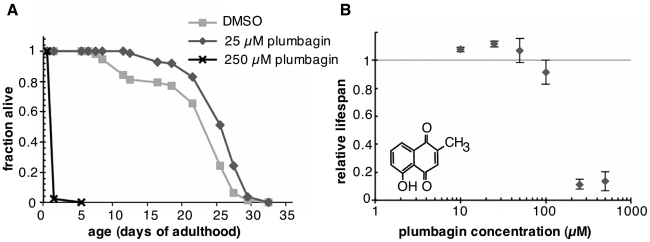
Low-dose plumbagin increases mean lifespan in C. elegans by stress hormesis. (A) Survival of wildtype adults treated with DMSO vehicle, 25 or 250 µM plumbagin. Mean lifespan was extended by 25 µM plumbagin (n = 137, p<0.0001) while the 250 µM dose was toxic and shortened lifespan (n = 144, p<0.0001). (B) Dose-response curve for mean survival relative to plumbagin concentration (µM). The plot shows an inverted U-shaped curve characteristic of stress hormesis. Error bars indicate variation in two to four independent lifespan experiments per dose. Individual trial data are presented in Table S1.

The microbial environment can be a source of stress that shortens *C. elegans* lifespan [24], [25]. Therefore, all subsequent experiments were conducted in the presence of 5-fluoro-2′-deoxyuridine (FUDR), a DNA synthesis inhibitor which is both bacteriostatic and bacteriocidal [26], [27]. In four trials with wild type *C. elegans*, treatment with 25 µM plumbagin was associated with an average increase in mean lifespan of 12+/−2% (p<0.0001, two-way ANOVA) (Fig. 1, Table S1). Treatment with 50 µM plumbagin was also associated with a significant increase of mean lifespan in two of three trials. These doses of plumbagin did not increase maximum lifespan. Plumbagin's prolongevity effect was lost at 100 µM and doses between 200–500 µM were toxic. Based on this screen, we conclude that plumbagin and juglone possessed hormetic activity that could extend lifespan in *C. elegans*. We did not detect beneficial effects from the other phytotoxins tested, although we cannot rule out the possibility that we failed to test the appropriate doses for hormesis.

### Low concentrations of plumbagin extend *C. elegans* lifespan through stress-responsive transcription factors

**Figure 2:**
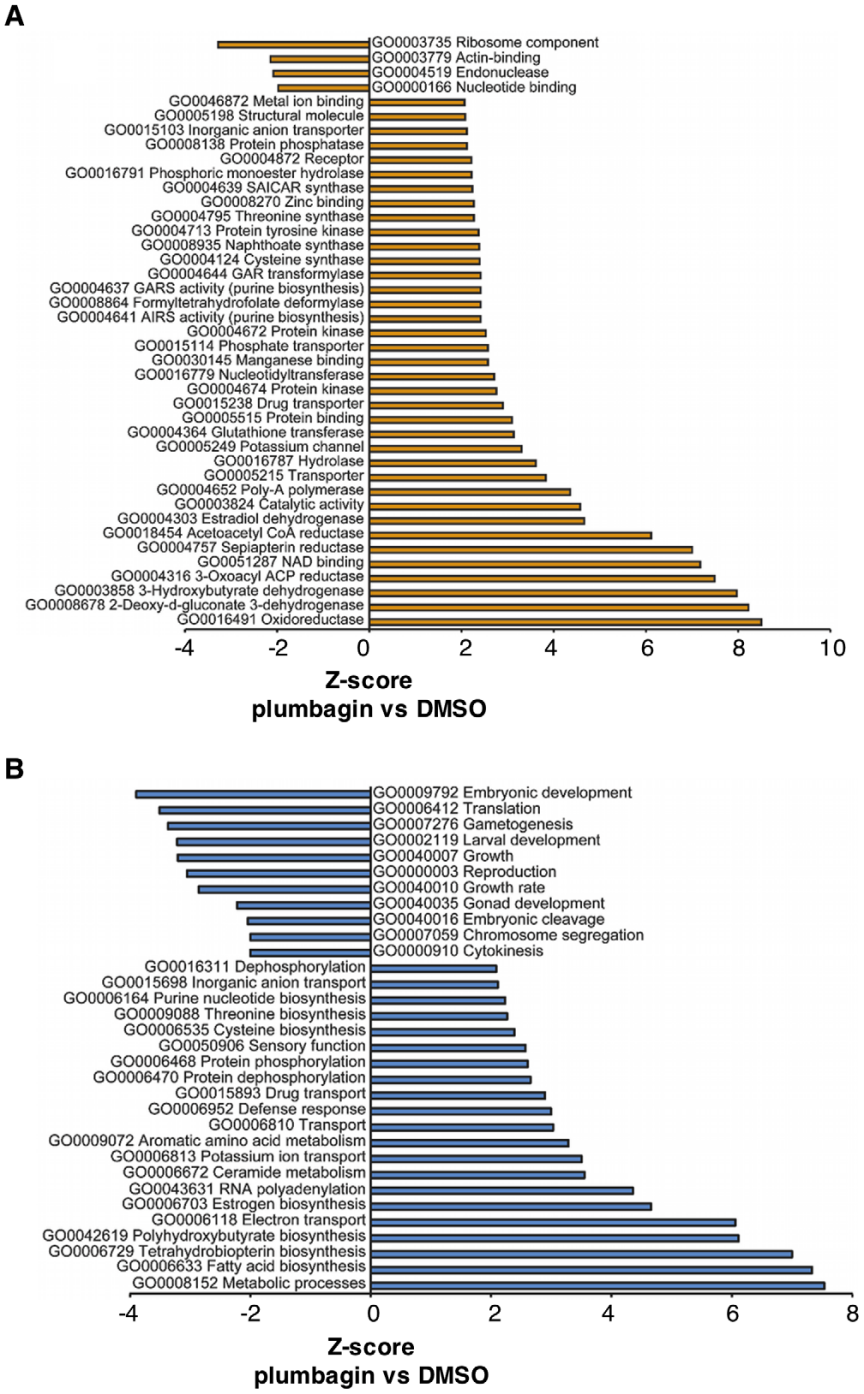
Transcriptional effects of plumbagin under non-toxic conditions in *C. elegans*. (A,B) Results of GO analysis of molecular function (A) and biological processes (B) for transcripts differently expressed in animals treated with plumbagin versus vehicle (DMSO). Results indicate upregulation of genes involved in oxidative stress resistance functions (A) and biological functions related electron transport (B), possible related to electron capture. Furthermore, reductions in growth and developmental processes were identified in plumbagin-treated animals, consistent with a shift of resources to stress response.

To identify pathways activated by plumbagin, we performed microarray analysis of *C. elegans* gene expression after a 2-day treatment with 100 µM plumbagin. This dose is slightly greater than that required for stress hormesis but is only weakly toxic to adult *C. elegans* (0%, −11%, −16% in 3 trials, Table S1). We chose this dose with the goal of maximizing the expression level of plumbagin-responsive genes. GO analysis revealed that the strongest effect of plumbagin was upregulated expression of oxidoreductase activity, consistent with plumbagin's action as an oxidative stressor (Fig. 2A) [28], [29]. Additionally, plumbagin treatment was associated with repression of growth, development and reproductive processes, indicating a shift from energy expenditure to energy conservation and repair (Fig. 2B). Plumbagin treatment led to the upregulation of several genes for glutathione-S-transferases, cytochrome P450 enzymes and small-molecule dehydrogenases, as was previously observed for juglone and hyperbaric oxygen in *C. elegans* [30], [31].

CNC-family NF-E2 transcription factors direct antioxidant enzyme expression as part of the Phase 2 stress response, an important pathway for cellular oxidative stress resistance [32]. Plumbagin activates NF-E2 target genes in human neuroblastoma cells [21]. Therefore, the *C. elegans* CNC transcription factor, SKN-1, is one candidate for mediating the beneficial effects of plumbagin. Our microarray analysis surveyed 14 of the top 15 upregulated *skn-1* targets [33]. Plumbagin treatment increased expression of 9 of these *skn-1* targets by 2-fold or greater (Table 2). A second candidate effector for plumbagin's benefits is the stress-responsive FoxO transcription factor, DAF-16. In *C. elegans*, stresses such as growth factor withdrawal, starvation, oxidative and heat stress, trigger DAF-16/FoxO nuclear translocation and expression of target genes [14], [34]–[37]. In C. elegans, lifespan extension by stress hormesis from juglone required *daf-16* activity [19]. The activities of *skn-1* and *daf-16* are interdependent for lifespan regulation [38]. Among 38 upregulated *daf-16* target genes [39] surveyed in our experiment, only two were upregulated with 100 µM plumbagin (Table 3). Plumbagin did not alter expression of sod-3, a direct target of DAF-16 [35].

**Table 2:** Effect of plumbagin on expression of *skn-1* targets.

Sequence ID	Gene name	skn-1 target *	Plumbagin treatment	
			Fold-change	P
K08F4.7	*gst-4*	√	4.9	<0.001
C32H11.4		√	2.1	0.001
C32H11.12	*dod-24*	√	2.0	0.006
ZK1058.6	*nit-1*	√	2.5	<0.001
Y45G12C.2	*gst-10*	√	2.3	0.01
K10D11.1	*dod-17*	√	2.7	<0.001
C32H11.3		√	1.9	0.01
Y102A11A.3		√	−1.1	0.89
T26C5.1	*gst-13*	√	1.8	0.05
F56D5.3		√	3.8	<0.001
K10C2.3		√	1.7	<0.001
F23B2.12	*pcp-2*	√	1.1	0.53
F55G11.2		√	2.0	<0.001
C35B1.5		√	2.0	0.02

Average fold-change from 4 biological replicates comparing gene expression in animals treated with 100 µM plumbagin vs DMSO vehicle control using *C. elegans* 4x44K oligo microarrays (Agilent Technologies, USA).

*14 *skn-1* targets (Oliveira et al. 2009).

**Table 3:** Effect of plumbagin on expression of *daf-2* pathway targets.

Sequence ID∧	Gene name	Plumbagin treatment		Sequence ID∧	Gene name	Plumbagin treatment	
		Fold-change	P			Fold-change	P
C55B7.4	*acdh-1*	−1.2	0.39	F38E11.2	*hsp-12.6*	-1.2	0.24
C46F4.2	*acs-17*	1.0	0.64	T27E4.8	*hsp-16.1*	1.1	0.57
F32A5.5	*aqp-1*	1.2	0.02	Y46H3A.3	*hsp-16.2*	1.0	0.55
K11D2.2	*asah-1*	1.0	0.54	C02A12.4	*lys-7*	−3.4	0.002
H22K11.1	*asp-3*	−1.6	0.13	C17G10.5	*lys-8*	1.2	0.68
C50B8.2	*bir-2*	−1.1	0.60	R03E9.1	*mdl-1*	−1.6	0.15
Y54G11A.6	*ctl-1*	−1.3	0.04	K11G9.6	*mtl-1*	2.2	0.01
Y54G11A.5	*ctl-2*	−1.2	0.21	F49E11.9	*scl-1*	−1.6	0.76
T10B9.1	*cyp-13A4*	−1.2	0.06	F43D9.4	*sip-1*	1.0	0.47
B0213.15	*cyp-34A9*	−1.3	0.23	C08A9.1	*sod-3*	1.0	0.14
K07C6.4	*cyp-35B1*	−1.4	0.41	K12G11.3	*sodh-1*	1.4	0.003
K07E3.3.2	*dao-3*	−1.0	0.68	K12G11.4	*sodh-2*	1.8	<0.001
C24B9.9.1	*dod-3*	−1.7	0.14	T07C4.4	*spp-1*	−1.0	0.26
T20G5.7	*dod-6*	1.4	0.42	T22G5.7	*spp-12*	1.7	<0.001
F10D2.9	*fat-7*	−1.0	0.23	C06B3.4	*stdh-1*	1.2	0.33
C52E4.1	*gcp-1*	−1.0	0.36	F11A5.12	*stdh-2*	1.2	<0.001
C05E4.9	*gei-7*	1.2	0.48	F28D1.3	*thn-1*	−1.1	0.25
R12A1.4	*ges-1*	−1.2	0.97	AC3.7	*ugt-1*	1.0	0.17
T28B8.2	*ins-18*	−1.5	0.87				

∧ 37 *daf-2* pathway targets (Murphy et al. 2003).

**Figure 3:**
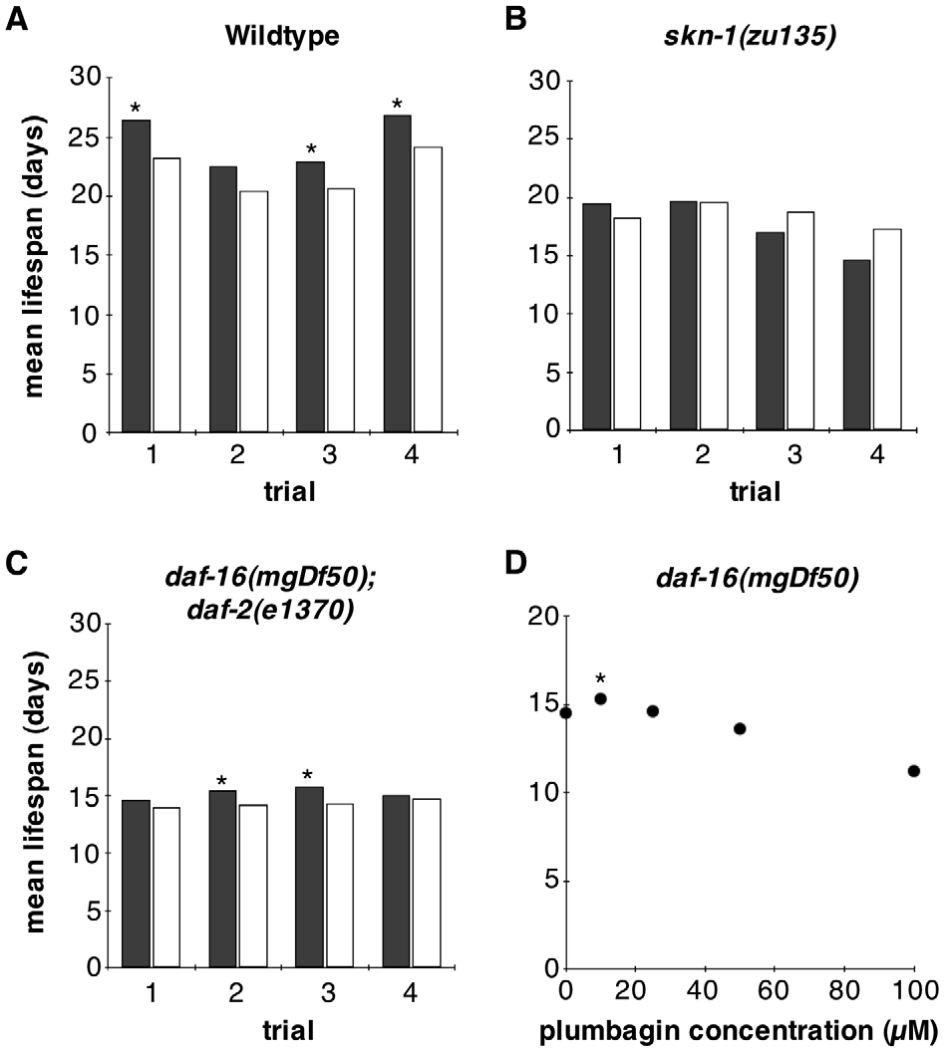
Effect of plumbagin on adult lifespan of *skn-1* and *daf-16* mutants. (A–C) Mean adult lifespan on 25 µM plumbagin (filled columns) compared to vehicle control (unfilled columns) was measured in 4 independently-conducted trials for (A) wildtype, (B) *skn-1(zu135)* and (C) *daf-16(mgDf50); daf-2(e1370)* adults. Asterisks mark trails in which mean adult lifespan was extended by 25 µM plumbagin treatment with Log-Rank probability > 0.01. Lifespan data for additional plumbagin doses is presented on Table S1. (D) Dose-response curve of *daf-16(mgDf50)* adult lifespan at 0, 10, 25, 50 and 100 µM plumbagin. Asterisk indicates a positive effect of plumbagin on lifespan with Log-Rank probability > 0.01. Data for lifespan trials is presented in Table S1.

Next, we examined whether lifespan extension by low-dose plumbagin required *skn-1* and *daf-16* activity. Consistent with a role for *skn-1*, 25 µM plumbagin did not extend lifespan of *skn-1(zu135)* animals in four independent trials (Fig. 3B). Additionally, *skn-1* RNAi abrogated lifespan extension from treatment with 10, 25 and 50 µM plumbagin (Table S2). Furthermore, *skn-1* RNAi enhanced toxicity of plumbagin at doses of 50 µM and above. Thus, *skn-1* activity was required both for the longevity benefits of plumbagin, and for normal resistance to plumbagin toxicity.

We also examined the effects of plumbagin on lifespan in both *daf-16(mgDf50);* *daf-2(e1370)* and *daf-16(mgDf50)* backgrounds lacking *daf-16* activity (Fig. 3C, D). Lifespan was extended by plumbagin treatment in these *daf-16*-deficient backgrounds, although the effect was significantly weaker and more variable than in the wildtype background. Together, these results implicate both *skn-1* and *daf-16* as mediators of lifespan extension by low-doses of plumbagin.

### A GFP reporter for a SKN-1 transcriptional target is a dose-responsive biosensor of plumbagin exposure

**Figure 4:**
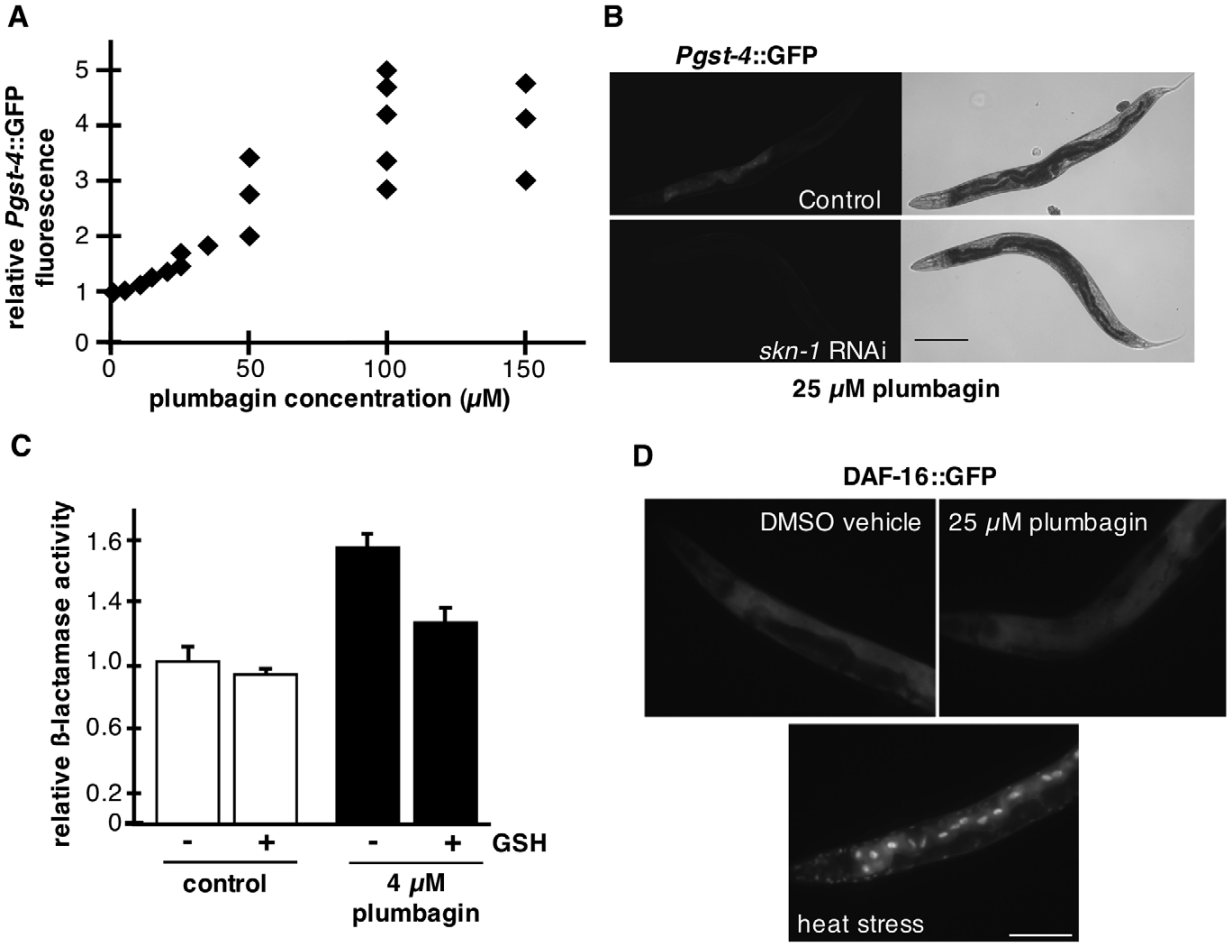
Effect of low-dose plumbagin on DAF-16, SKN-1 and ARE transcriptional reporters in *C. elegans* and HepG2 cells. (A) Dose-response curve for *Pgst-4*::GFP fluorescence versus plumbagin concentration. Levels of *Pgst-4*::GFP were determined for a minimum of 20 worms per condition per trial by average background-subtracted values for whole worm fluorescence of treated animals normalized to controls; p<0.0001 (T-test) in every trial at doses of 20 µM plumbagin and above. Each data point represents a single trial. Results for individual trials are shown in Table S2. (B) Representative images of *Pgst-4*::GFP in wildtype day 1 adults treated for two days with 25 µM plumbagin under control conditions (left panel) or with skn-1 RNAi (right panel). In control animals, 25 µM plumbagin was associated with increased *Pgst-4*::GFP in the intestine and muscles and skn-1 RNAi abrogated the increases in *Pgst-4*::GFP fluorescence levels. Bar, 0.2 mm. (C) ARE reporter activity in HepG2 cells treated with DMSO vehicle control (open) or 4 µM plumbagin (shaded). Plumbagin activated ARE reporter beta-lactamase expression. Co-incubation with reduced glutathione significantly reduced induction of the ARE reporter by plumbagin. Data are relative beta-lactamase activity + SEM. (D) Adult TH356 hermaphrodites expressing a DAF-16::GFP translational fusion were treated as indicated and localization of DAF-16::GFP monitored as a measure of DAF-16 activation. Thermal stress (37°C, 20 minutes) induced dramatic nuclear accumulation of DAF-16::GFP in intestinal nuclei. In contrast, DMSO vehicle and 25 µM plumbagin treatments failed to induce nuclear DAF-16::GFP accumulation. Two independent experiments were performed with n = 20 animals/treatment. For DMSO and plumbagin, animals were transferred to treatments and DAF-16:GFP localization was scored after 3, 24 and 48 hours. Shown are representative 48-hour treatments. Bar, 0.1 mm; all images were collected using identical exposure times.

Using GFP reporters, we examined whether activation of *skn-1* or *daf-16* by low-dose plumbagin could be monitored in vivo. *C. elegans* strain CL2166 carries a GFP reporter expressed from the *gst-4* promoter [40]. The *gst-4* promoter contains two predicted SKN-1 binding sites and *Pgst-4*::GFP expression induced in response to oxidative stress from hyperbaric oxygen and hydrogen peroxide was *skn-1*-dependent [41]. Treatment with 25 µM plumbagin induced expression of *Pgst-4*::GFP in the intestine, with weaker expression in the head and body muscles (Fig. 4B). We quantified *Pgst-4*::GFP fluorescence in the bodies of animals treated for 48 hours with 0-150 µM plumbagin, encompassing a range of beneficial and toxic doses. The increase in *Pgst-4*::GFP was approximately linear between 0-50 µM plumbagin and appeared to become saturated, but also variable, at higher plumbagin doses (Fig. 4A, Table S2). In three trials, the beneficial dose of 25 µM plumbagin was associated with a 57+/−13% increase in *Pgst-4*::GFP fluorescence over background (p<0.001, T-test). 50 µM plumbagin induced a range of *Pgst-4*::GFP levels, with whole worm fluorescence increased by 96%, 173%, and 241% compared to controls in three trials. We note that 50 µM plumbagin increased lifespan in a subset of trials, suggesting that this concentration may represent the upper end of the beneficial dose range. *Pgst-4*::GFP induction by plumbagin was *skn-1*-dependent. In animals treated with *skn-1* RNAi, plumbagin failed to induce *Pgst-4*::GFP expression in most tissues (Fig. 4B; Table 4). The idea that there is an optimal window of *skn-1* activity for lifespan extension is consistent with data showing that moderate over-expression of *skn-1* extended lifespan while high-copy over-expression of *skn-1* was toxic [38].

**Table 4:** Effect of *skn-1* RNAi on *Pgst-4*::GFP induction by plumbagin.

Expt.	Plumbagin (µM)	RNAi	AMPI ∧	Std. Dev.	Ratio †	P *	n
1	0	L4440	8.2	2.0	−	−	30
		*skn-1*	3.7	0.5	0.46	<.001	30
	25	L4440	12.9	2.2	1.57	<.001	20
		*skn-1*	4.3	0.6	0.52	<.001	20
2	0	L4440	5.3	0.9	−	−	34
		*skn-1*	2.7	0.4	0.52	<.001	34
	100	L4440	32.2	8.6	6.08	<.001	32
		*skn-1*	4.4	0.7	0.84	<.001	36
3	0	L4440	5.8	0.9	−	−	33
		*skn-1*	2.7	0.4	0.46	<.001	33
	100	L4440	23.0	5.1	3.94	<.001	22
		*skn-1*	4.8	1.2	0.82	<.01	20

Animals (CL2166) were raised from embryo to adulthood on HT115 bacteria containing either the L4440 RNAi vector or a plasmid inducing *skn-1* RNAi. Adults were treated for two days with plumbagin or DMSO vehicle control before GFP visualization.

∧ AMPI represents the average whole worm background subtracted mean pixel intensity for the treatment group.

† AMPI ratio for treatment relative to the L4440 vehicle (DMSO) control.

T-test compares the AMPI of each worm in the treatment group with those of the control (0 µM plumbagin, L4440) in that experiment.

In cultured mammalian neurons, plumbagin activated expression of target genes containing the antioxidant response element (ARE) in an Nrf2-dependent manner [21]. Nrf2 activity is induced in response to oxidative stress and plumbagin has reported ROS generating activity [42]. We therefore examined whether plumbagin activation of ARE-containing targets could be altered by treatment with glutathione, an antioxidant capable of scavenging plumbagin-generated ROS. Indeed, ARE-reporter induction by plumbagin was significantly diminished by cotreatment with glutathione (Fig. 4C). These data support the hypothesis that plumbagin activates Nrf2 target gene expression through alteration of the cellular redox environment.

In two independent experiments with 20 animals each, 25 µM plumbagin failed to increase nuclear localization of a DAF-16::GFP reporter (Fig. 4D). Therefore, we utilized the *Pgst-4*::GFP for a biosensor of stress hormesis in all subsequent experiments.

### Analysis of plumbagin analogs for improved benefit:toxicity profiles

Two problems for translating interventions based on stress hormesis mechanisms are the limited dose range for beneficial effects and toxicity at higher doses. Therefore, we investigated whether plumbagin analogs existed which could activate *skn-1* and extend *C. elegans* lifespan without significant toxicity at higher doses. A group of six plumbagin analogs, consisting of 3 naphthoquinones and 3 tetralones, was screened for activation of *Pgst-4*::GFP and effects on lifespan in *C. elegans*. Tetralones, which lack the 4-ketone of plumbagin, did not activate *Pgst-4*::GFP fluorescence (Table S3) and conferred no survival benefit at any tested dose (Table S1). In addition, the tetralones were generally non-toxic to *C. elegans* at concentrations up to 500 µM.

**Figure 5:**
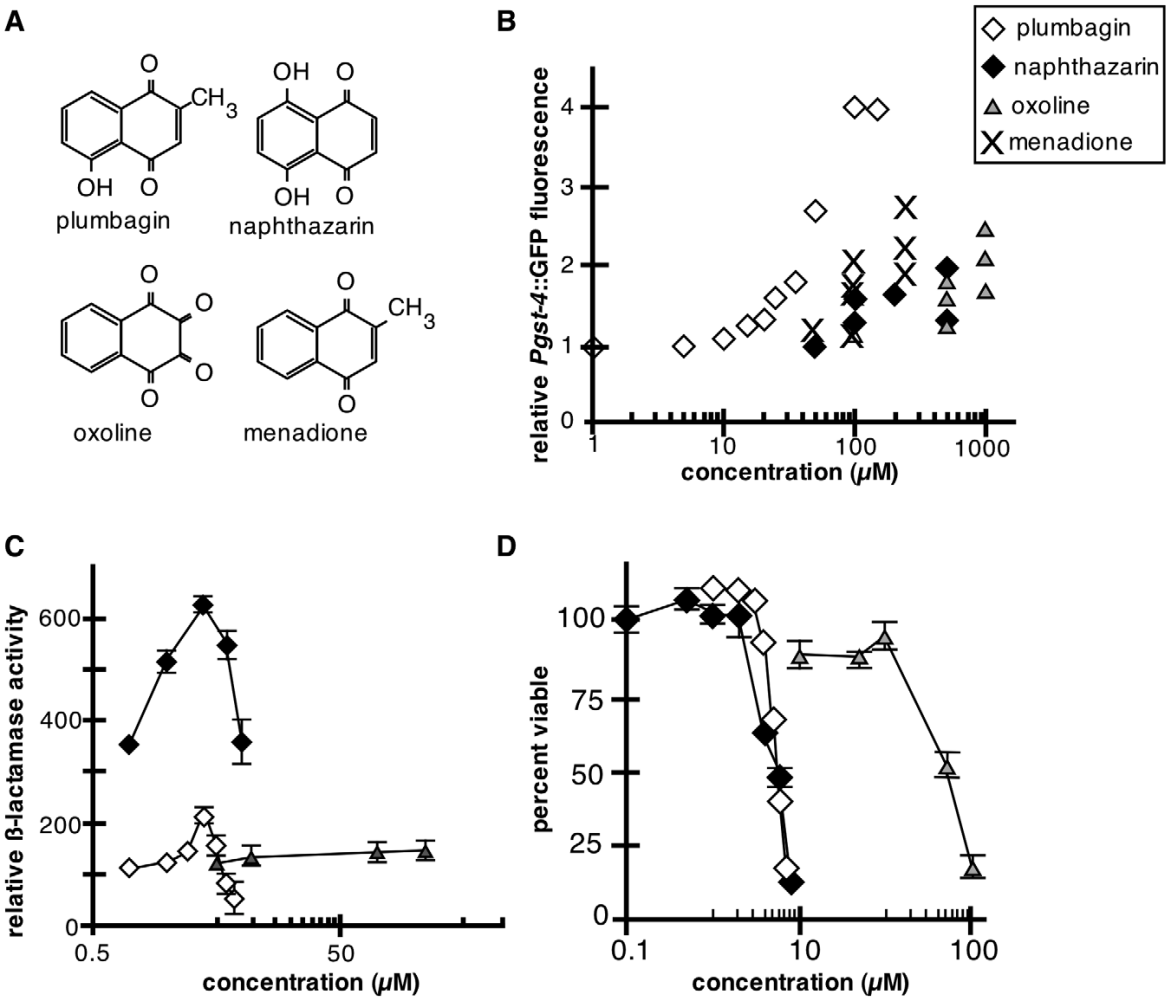
Effects of naphthazarin, oxoline and menadione on *skn-1* and Nrf2 targets in *C. elegans* and HepG2 cells. (A) Structures of plumbagin, naphthazarin, oxoline and menadione. (B) Induction of *Pgst-4*::GFP by naphthazarin, oxoline, and menadione. Data are results from independent experiments measuring whole worm *Pgst-4*::GFP fluorescence compared to vehicle-treated controls (Table S3). For comparison, the average level of *Pgst-4*::GFP fluorescence in plumbagin-treated animals is shown on the same plot (diamonds, data shown on Fig. 4A). Shaded diamonds, naphthazarin, n≥30, p<0.0001 for 100 µM and above; triangles, oxoline, n≥16, p<0.0001 for 500 µM and above; crosses, menadione, n≥18, p<0.0001 for 100 µM and above. (C) Plumbagin (diamonds) and naphthazarin (shaded diamonds) activated ARE reporter expression (beta-lactamase) in HepG2 cells. Toxicity at higher concentrations of plumbagin and naphthazarin resulted in decreased reporter gene expression. Oxoline (triangles) failed to induce expression of ARE driven beta-lactamase. The ARE reporter cell line HepG2 was exposed to the indicated concentrations of plumbagin, naphthazarin or oxoline and ARE-driven beta-lactamase activity was assessed 24 hrs after exposure. Data are mean beta-lactamase activity ± SEM (n = 3−4). (D) HepG2 cells were treated with plumbagin (diamonds), naphthazarin (shaded diamonds) or oxoline (triangles) at indicated concentrations. Cell viability was assessed 24 hrs after exposure. Results are the mean from 3−4 experiments +/− SEM.

In contrast to the tetralones, all three naphthoquinones, menadione, naphthazarin and oxoline, increased *Pgst-4*::GFP expression, although not as strongly as plumbagin (Fig 5A, B; Table S3). As with plumbagin, increases in *Pgst-4*::GFP expression in worms treated with naphthazarin and oxoline were abrogated by skn-1 RNAi (data not shown). Plumbagin, but not menadione, was previously shown to induce the Nrf2-mediated Phase 2 response in cultured mammalian cells [21]. We therefore compared activation of the Nrf2-dependent ARE reporter by naphthazarin, oxoline and plumbagin in HepG2 cells. Both plumbagin and naphthazarin induced the ARE reporter, while oxoline failed to activate the ARE reporter at up to 100-fold higher concentrations (Fig. 5C). We also examined survival of HepG2 cells in the presence of each naphthoquinone. Plumbagin and naphthazarin had similar toxicity profiles, while oxoline was approximately 100-fold less toxic than plumbagin (Fig. 5D).

We considered the possibility that naphthoquinones extended lifespan by protective effects against bacterial proliferation. The classical approach for testing this possibility is to measure lifespan in worms fed on a lawn of dead or growth-inhibited bacteria. As mentioned, all lifespan assays were conducted in the presence of FUDR, which is bacteriostatic and bacteriocidal. Consistently, average lifespan was longer when using FUDR-containing medium as compared to medium lacking FUDR (20.2 days vs 15.9 days, respectively; p<0.0001, Log-Rank). This eliminated the possibility that plumbagin, naphthazarin and oxoline extended lifespan by simply inhibiting bacterial growth. We further probed this question by testing the effects of these drugs in the presence of heat-killed bacteria. However, these experiments were confounded by the fact that naphthoquinone toxicity was significantly higher in the presence of heat-killed bacteria (Table S4). There are several explanations for this result. One is that bacteria were protective against naphthoquinone toxicity which was necessary to unmask the hormetic effects in *C. elegans*. Thus, growth on heat-killed bacteria could have shifted the hormetic dose range to doses that were much lower than those we tested. An alternative explanation is that bacteria enzymatically converted the naphthoquinones into products with hormetic activity in *C. elegans*. We did not further investigate these possibilities.

### Naphthazarin and oxoline extended *C. elegans* lifespan through different mechanisms

**Figure 6:**
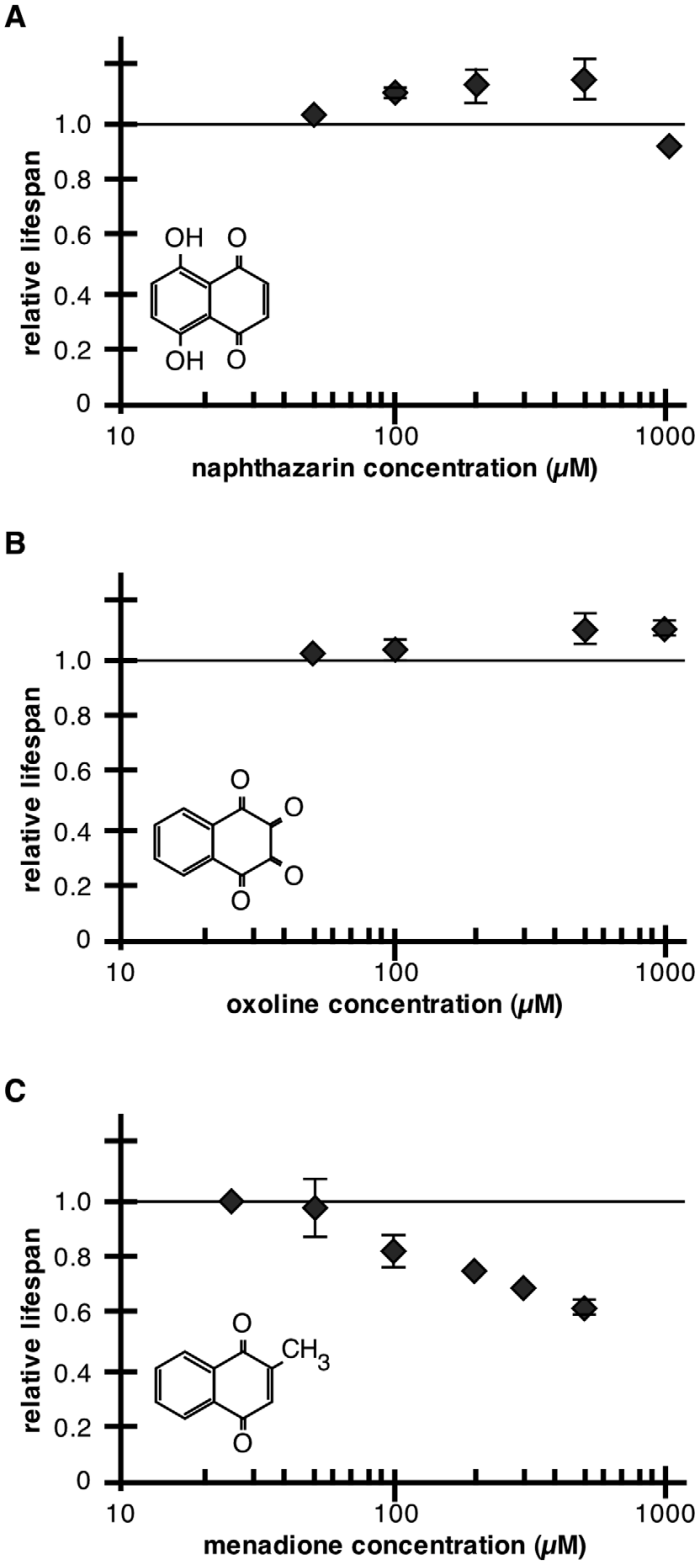
Effects of naphthazarin, oxoline, and menadione on *C. elegans* survival. (A–C) Dose-response relationship of relative *C. elegans* survival with respect to naphthoquinone concentration. Chemical structures for each compound are shown on each plot. (A) Naphthazarin increased *C. elegans* lifespan at higher doses than plumbagin. Data represent one trial at 50 µM (n = 67, p = 0.2), and three trials each at 100 µM (n≥93, p<0.0001 in 2 of 3 trials), 200 µM (n≥96, p<0.0001 in 3 of 3 trials), and 500 µM (n≥66, p<0.0001 in 2 of 3 trials). (B) Oxoline treatment extended mean lifespan at doses of 500 µM (n≥95, p<0.0001 in 3 of 4 trials) and 1 mM (n≥61, p<0.0001 in 2 of 2 trials). (C) Menadione treatment shortened *C. elegans* lifespan and did not provide a significant lifespan benefit at any tested dose (13 trials at various concentrations with n≥59 per condition, p<0.0001 in all trials at 100 µM and above).

We found that naphthazarin and oxoline, but not menadione, could extend *C. elegans* lifespan. At doses between 100–500 µM, naphthazarin treatment was associated with increased mean and maximum lifespan (e.g., 200 µM, 13+/−6% increase in mean lifespan, P<0.0001 in each of three trials) (Fig. 6A; Table S1). For oxoline, a 500 µM dose extended mean lifespan by 13–15% in 3 of 4 trials and increased maximum lifespan by at least 10% in all 4 trials. 1 mM oxoline was also associated with significant increases in mean and maximum lifespan (Fig. 6B; Table S1). Comparison of relative *Pgst-4*::GFP fluorescence and lifespan indicated that beneficial concentrations of naphthazarin, oxoline and plumbagin were associated with 50–150% increased *Pgst-4*::GFP expression (Table 5). However, *Pgst-4*::GFP expression was not strictly correlated with lifespan extension, as menadione, which also induced *Pgst-4*::GFP within this range, had no effect on lifespan at low concentrations and was toxic at high concentrations (Fig. 6C). This may reflect additional toxic effects of menadione *in vivo*.

**Table 5:** Effects of plumbagin, naphthazarin and oxoline on *Pgst-4*::GFP and lifespan in wildtype *C. elegans*.

Dose (µM)	Plumbagin		Naphthazarin		Oxoline	
	Ratio *Pgst-4*::GFP*	Ratio lifespan†	Ratio *Pgst-4*::GFP*	Ratio lifespan†	Ratio *Pgst-4*::GFP*	Ratio lifespan†
1	0.94	1.01				
2.5		1.03				
5	0.97	1.03				
7.5		1.00				
10	1.06	1.08				
15	1.23					
20	1.29					
25	1.57	1.12				
35	1.79					
50	2.70	1.07	1.01	1.02		1.02
100	4.00	0.91	1.43	1.10	1.17	1.04
150	3.97	0.77				
200		0.16	1.63	1.12		
250		0.12				
300		0.14				
400		0.10				
500		0.14	1.63	1.15	1.54	1.11
1000					2.07	1.12

*Ratio of *Pgst-4*::GFP fluorescence in animals treated with indicated dose versus vehicle. Data for individual trials is presented in Table S3.

† Ratio of adult lifespan in animals treated with indicated dose versus vehicle. Data for individual trials is presented in Table S1.

**Figure 7:**
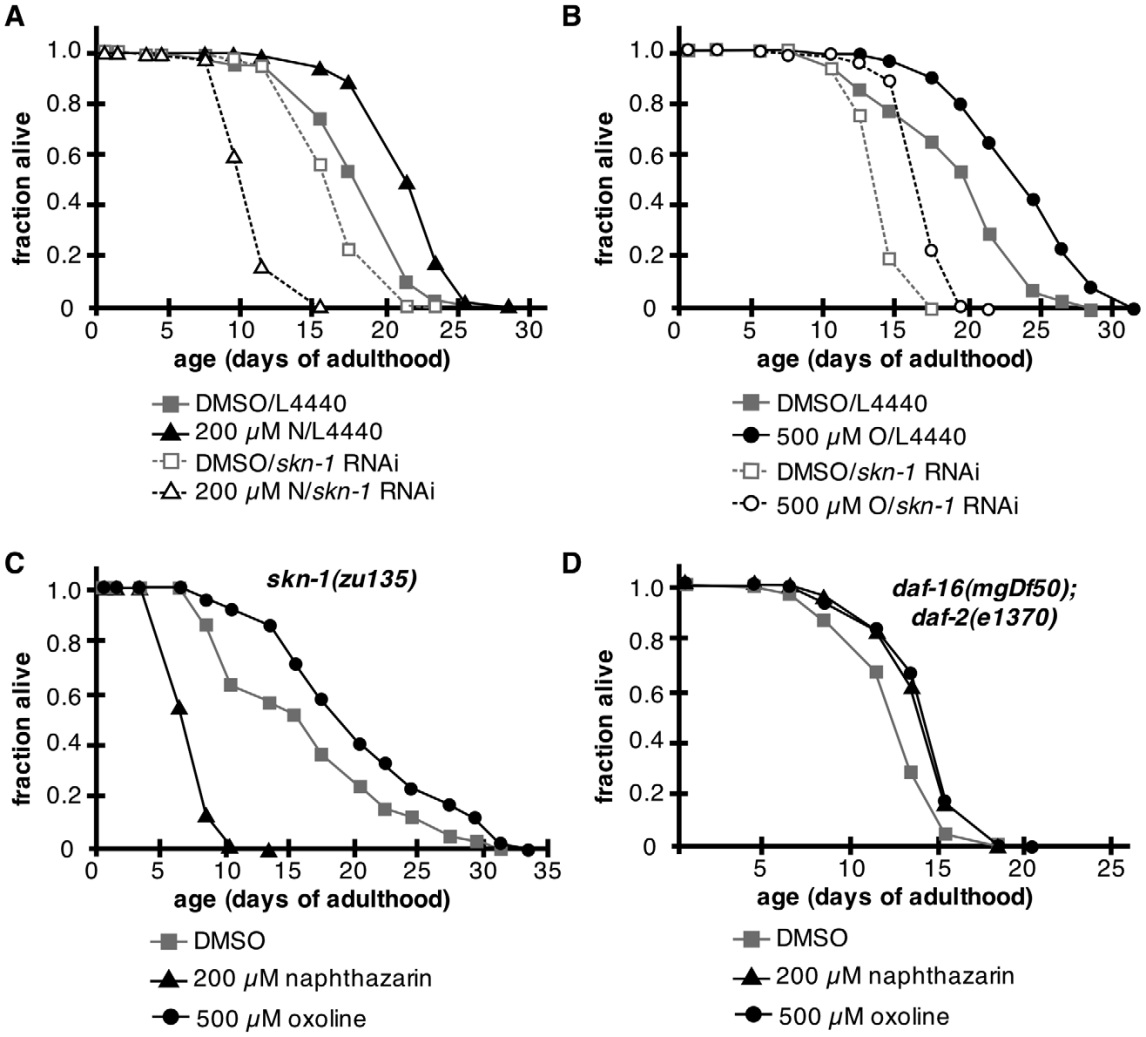
Requirement for skn-1 in lifespan extension by naphthazarin. (A) *skn-1* RNAi abrogated lifespan extension by 200 µM naphthazarin. Filled squares, L4440 treated with DMSO vehicle control (n = 80); open squares, *skn-1* RNAi treated with DMSO vehicle control (n = 125); filled triangles, L4440 treated with 200 µM naphthazarin (n = 88, p<0.0001); open triangles, *skn-1* RNAi treated with 200 µM naphthazarin (n = 127, p<0.0001). (B) 500 µM oxoline was associated with similar increases in lifespan for both L4440 and *skn-1* RNAi treated groups. Filled squares, L4440 treated with DMSO control (n = 117); open squares, *skn-1* RNAi treated with DMSO vehicle control (n = 131); filled circles, L4440 treated with 500 µM oxoline (n = 106, p<0.0001); open circles, *skn-1* RNAi treated with 500 µM oxoline (n = 124, p<0.0001). (C) Lifespan of *skn-1(zu135)* adults was shortened by 200 µM naphthazarin (triangles, n = 110, p<0.0001) relative to DMSO vehicle control (squares, n = 123), but lengthened by 500 µM oxoline (circles, n = 117, p<0.0001). (D) Mean lifespan of *daf-16(mgDf50); daf-2(e1370)* adults was extended by 200 µM naphthazarin (triangles, n = 116, p<0.0001) and 500 µM oxoline (circles, n = 121, p<0.0001), relative to DMSO vehicle treated controls (squares, n = 80).

Because beneficial doses of napthazarin and oxoline could induce *skn-1*-dependent *Pgst-4*::GFP expression at levels similar to those induced by beneficial doses of plumbagin, we tested whether lifespan extension by oxoline and naphthazarin were also *skn-1* dependent. Animals treated with *skn-1* RNAi lived shorter on 200 µM naphthazarin than controls, with an average 10% decrease in mean and maximum lifespan over 4 trials (Fig. 7A; Table S2). The decrease was more severe in *skn-1(zu135)* mutants, in which lifespan was only 55+/−10% of controls in four trials (Fig. 7C; Table S1). These results indicate that lifespan extension by naphthazarin was dependent on *skn-1*, and that *skn-1* activity was protective against naphthazarin toxicity. In contrast, *skn-1* RNAi did not block lifespan extension by oxoline in 3 of 4 trials (Fig. 7B; Table S2). Additionally, 500 µM oxoline increased mean lifespan by 12+/−8% in *skn-1(zu135)* (Fig. 7C; Table S1). The finding that oxoline influenced lifespan independently of skn-1 is consistent with the finding that oxoline did not induce the ARE reporter in HepG2 cells. In *daf-16(mgDf50);daf-2(e1370)* animals, both 200 µM naphthazarin and 500 µM oxoline increased mean lifespan by 11+/−2%, indicating that lifespan extension by both compounds was independent of *daf-16* activity (Fig 7D; Table S1).

## Discussion

Our findings and those of previous studies [43], [44] suggest that interventions activating stress response pathways can reduce cellular damage, thereby slowing aging and increasing lifespan. Phytochemicals that exert cytoprotective effects, such as curcumin [45], [46] and sulforaphane [47], [48], do so by inducing the Phase 2 detoxification response via NF-E2 transcription factors. Here, we characterized the ability of three naphthoquinones, plumbagin, naphthazarin and oxoline, to extend *C. elegans* lifespan through stress hormesis pathways linked to the antioxidant response. In our study, lifespan extension by plumbagin and naphthazarin was dependent on *skn-1* activity. In the absence of skn-1 activity, the hormetic benefits of plumbagin were lost. In *C. elegans*, lifespan extension from stress hormesis by thermal stress and juglone-induced oxidative stress also require *daf-16* [19], [49]. Lifespan extension by plumbagin was weaker and more variable in *daf-16* mutants than in wildtype animals, suggesting that *daf-16* is a component of the hormetic pathway induced by plumbagin. However, we did not detect *daf-16* target gene induction or DAF-16::GFP nuclear localization by hormetic doses of plumbagin. Reduced insulin/IGF-1-like signaling (IIS) from the DAF-2/IIS receptor increases nuclear accumulation of SKN-1 and activates a subset of skn-1 dependent genes (including *gst-4*) independently of DAF-16 [38]. The interactions between *daf-16* and skn-1 complicate efforts to tease apart their individual roles in this pathway. For this reason, further study will be necessary to clearly define the daf-16's role in lifespan extension by low-dose plumbagin.

One aim of this study was to identify derivatives of a hormetic chemical that display broad beneficial dose ranges and lower toxicity. Such properties would be necessary in order to consider clinical translation of hormetic interventions. By comparing the relative effects of plumbagin on longevity and *Pgst-4*::GFP expression, we predicted that compounds which increased *Pgst-4*::GFP expression by 50-150% above basal levels would also extend lifespan. Plumbagin doses that activated expression above this level were toxic, possibly due to excessive levels of oxidative stress and cellular damage. We identified three naphthoquinones, naphthazarin, oxoline and menadione, that could induce *Pgst-4*::GFP. One of these, naphthazarin, behaved similarly to plumbagin with a broader beneficial dose range, as desired. Naphthazarin extended lifespan at doses which induced *Pgst-4*::GFP expression to the same range as hormetic doses of plumbagin. Overall, naphthazarin was less potent than plumbagin in *C. elegans*.

Although menadione induced *Pgst-4*::GFP within the predicted beneficial range, it failed to extend lifespan at any concentration tested. One explanation is that menadione affects cells through multiple mechanisms, which overlap partially with mechanisms for plumbagin toxicity. Findings in *Saccharomyces cerevisiae* are consistent with this idea. Low-dose pretreatment with either plumbagin or menadione induced adaptive responses protective against plumbagin toxicity, while low-dose plumbagin failed to protect from menadione toxicity [50]. These data are consistent with the idea that menadione has additional toxic activities that may mask its potential benefits in *C. elegans*.

Oxoline, the third plumbagin derivative we studied, extended *C. elegans* lifespan through *skn-1*- and *daf-16*-independent mechanisms. Oxoline weakly activated *Pgst-4*::GFP and failed to activate ARE reporter expression in HepG2 cells. We found oxoline to be non-toxic to *C. elegans* and mammalian cells within the tested dose range. Oxoline belongs to the class of bacteriocidal quinolone antibiotics that inhibit DNA topoisomerase activity. Oxoline has been prescribed for treatment of urinary tract infections from gram-negative bacteria and is used for antiviral therapy [51], [52]. Based on this evidence, it is possible that oxoline affects *C. elegans* lifespan through antimicrobial effects.

Although the oxidative stress response is molecularly conserved between worms and mammals, we uncovered differences between these systems that call for caution when attempting to extrapolate effects of specific longevity interventions across species. For instance, oxoline induced *Pgst-4*::GFP expression in *C. elegans* but failed to induce an ARE reporter in mammalian cells. Moreover, sulforaphane and plumbagin had different relative effects on expression of ARE reporter and *Pgst-4*::GFP levels [21]. Numerous differences between *C. elegans* nematodes and cultured mammalian cells could affect the activities of specific compounds in each system. First, compounds are likely to enter cultured cells more easily than *C. elegans* adults, which are surrounded by an impermeable cuticle. Second, intact *C. elegans* may possess a distinct array of metabolizing enzymes from cultured cells which could alter the production of bioactive metabolic intermediates. Third, the systems differ in mechanisms for excreting compounds from the cellular cytoplasm. Finally, the culture conditions for nematodes and cells are different and expose the compounds to significantly different environments.

We propose that *C. elegans* provides a facile approach to identifying families of compounds that can activate targets with conserved anti-aging or stress response functions. However, individual compounds within such families may have distinct activities reflecting differences in uptake, metabolism and environmental conditions. Ideally, a group of related compounds should be compared to identify those with maximal effectiveness in a particular experimental system or therapeutic context. In this light, our findings positively demonstrate that stress hormesis mechanisms can be accessed for longevity benefits in a whole organism, such as *C. elegans*, and that structural analogs of hormetic compounds may have improved toxicity profiles. However, care must be taken when extrapolating findings about specific compounds between experimental systems.

## Materials and Methods

### Chemicals

The following chemicals were obtained commercially, as indicated: plumbagin (#P7262), naphthazarin (#388513), menadione (Fluka #67900), visnagin (#254932), eugenol (#35995), farnesol (#F203), alpha-asarone (#231282), piperine (#P49007), L-canavanine (#C1625), domoic acid (#D6152), precocene II (#194913), sesamin (sesamol, #S8518), juglone (#H47003), 5-hydroxy-1-tetralone (#219975) and 5,8- dimethoxy-1-tetralone (#569658) were obtained from Sigma-Aldrich Co., St. Louis, MO; veratrine (#219834) was from MP Biomedicals, Solon, OH; anabasine (#L18484) was from Alfa Aesar, Ward Hill, MA and marmesan (#CDL 0080-0004) was from Chemical Diversity, San Diego, CA. Oxoline was obtained from Wako Chemicals, Richmond, VA (#328-67371). 7-hydroxy-1-tetralone was obtained from Milestone PharmaTech, New Brunswick, NJ (#2A-0017). For each, chemical characterization was performed to confirm the correct chemical structure and, for agents that proved less than 98% pure, recrystallization and purification procedures were performed to obtain high chemical purity. Chemicals were stored at −20°C as 100 mM stock solutions in DMSO. To prepare *C. elegans* media for experimental treatments, chemicals were added from stock solutions to molten NGM agar and then poured into 3.5-cm culture dishes for use, with the exception of the screen for hormetic chemicals.

### *C. elegans* strains and growth conditions

*C. elegans* strains Bristol N2 (wild-type), CL2166 (N2; *dvIs19 (Pgst-4::gfp; rol-6))*, TJ356 (N2; zIs356 (*daf-16::gfp; rol-6))*, EU31 (*skn-1(zu135)/DnT1)*, and CY312 (*daf-16(mgDf50);daf-2(e1370))* were utilized in this work. Strains were grown and maintained at 15°C or room temperature (22 to 24°C) on Nematode Growth Media (NGM) containing 2.1% agar with *E. coli* strain OP50 as a food source [53], except as indicated for RNAi experiments. Age-synchronized populations were obtained by collecting the eggs laid by fertile adults over 5 to 7 hours and allowing them to develop to the young adult stage before transfer to treatment plates.

### Cell culture methods

The stable Nrf2/ARE reporter cell line, ARE-bla HepG2, was purchased from Invitrogen (Carlsbad, CA, USA) and maintained in Dulbecco's Modified Eagle's Medium (DMEM) containing 10% dialyzed fetal bovine serum (FBS), 2 mM glutamine, 0.1 mM nonessential amino acids, 25 mM HEPES, 1% penicillin/streptomycin, and 5 µg/ml of blasticidin (Invitrogen) at 37°C in a humidified 5% CO2 atmosphere. Blasticidin was removed from the media during experimental treatments. For cell viability and ARE reporter assays, ARE-bla HepG2 cells were plated in 96-well plates and allowed to adhere overnight. Cell viability was assessed 24 hours after incubation with the indicated concentration of plumbagin, naphthazarin and oxoline by Celltiter 96® AQUEOUS One Solution reagent (Promega, Madison, WI, USA). Nrf2-transactivation was measured by beta-lactamase activity according to the manufacturer's instructions (Invitrogen). All experiments were performed in triplicate.

### Screen for hormetic prolongevity chemicals

For the initial screen, compounds were suspended to 100 mM in either water or EtOH. 20 µL of each stock solution, diluted in water to 150 µL immediately prior to use, was dropped onto the surface of 10 mL NGM agar plates supplemented with live OP50 bacterial food. Plates were incubated at room temperature for 2 hours to allow compounds to diffuse into the media to a final concentration of 200 µM. Approximately 40 sterile *fem-1(hc17)* adults, raised at 25°C, were suspended in water and dropped onto the surface of test plates. Survival was scored daily for the first week, then at 1–3 day intervals until all animals were dead. Survival assays were terminated as soon as an outcome became apparent relative to paired control populations treated with vehicle alone. Chemicals that were toxic at 200 µM were tested at 100 µM. If the compound was toxic at 100 µM, it was further tested at 60 µM, 30 µM and 10 µM. Compounds that were not toxic at 200 µM were tested at 300 µM, and, if toxic, further tested at 100 µM for possible beneficial effects. Vehicle controls (water or ethanol) were included for all concentrations tested.

### Lifespan analyses

In all lifespan assays, worms were grown on NGM agar until day 1 of adulthood, the first day following the L4-adult molt. Young adults were transferred to fresh medium supplemented with indicated compounds or 0.2% DMSO as a vehicle control. For most treatments, the final concentration of the DMSO vehicle was <0.2%. Lifespan assays were performed at room temperature (22–23°C) and survival was scored every 2–4 days as the ability to move in response to touch with a platinum wire. 200 µM FUDR was used in lifespan assays to prevent progeny production, except for experiments with *fem-1(hc17)* hermaphrodites, which are sterile and do not produce progeny [22], [54]. FUDR is also bacteriocidal and bacteriostatic. Maximum lifespan was calculated by the mean lifespan of the oldest 10% cohort in each experiment. Log-Rank P-values within experiments were calculated using the Survival/Reliability function in JMP 5.0.1.2 (SAS, USA). Significance probability P-values over multiple experiments with the same conditions were calculated with the GLM Procedure in SAS 9.2 (SAS, USA), with treatment and experiment number as the two independent factors for two-way ANOVA, and days of life as the dependent variable.

### Microarray Analysis

Approximately 1,000 age-synchronized young adults were treated for two days with either 100 µM plumbagin or DMSO as a vehicle control and then washed three times in M9 and flash frozen with dry ice. RNA was isolated with the Absolutely RNA Miniprep Kit (Agilent Technologies, USA). RNA concentration and quality was assessed using an Agilent Bioanalyzer 2100 (Agilent Technologies, USA). Microarray hybridizations were performed with the *C. elegans* 4x44K Oligo Microarray (Agilent Technologies, USA). Raw microarray hybridization intensity data from four separate experiments were log-transformed and normalized to generate z-scores and subsequent z-ratios [55].

### RNA interference

RNA interference (RNAi) was induced by providing a food source of HT115 bacteria expressing double-stranded RNA corresponding to the targeted gene [56]. The RNAi medium was NGM agar supplemented with 100 µg/mL ampicillin and 1 mM isopropyl β-D-thiogalactoside (IPTG) to induce bacterial expression of the dsRNA trigger. Young adult animals (Po) were placed onto RNAi plates for several hours to lay eggs and then removed. Larvae developed to young adulthood on RNAi plates and were then transferred to fresh RNAi plates supplemented with test compounds or vehicle (DMSO). In this way, worms continued feeding on dsRNA-expressing bacteria throughout the experiment.

### Analysis of *Pgst-4*::GFP expression

Young CL2166 adults were transferred onto NGM agar supplemented with test compounds or vehicle (DMSO). After two days at room temperature (22–24°C), animals were mounted on 2% agarose pads and immobilized with 20 mM levamisole. Using a Nikon E900 microscope (Nikon Corporation, Japan), GFP fluorescence was visualized with a mercury illumination source at 100× magnification (10X objective, 10X ocular magnification). Images were captured using Openlab software, v. 5.5.0 (Improvision Inc., USA) controlling a Hamamatsu Orca CCD camera (Hamamatsu Photonics, Japan). Image processing was performed using Adobe Photoshop CS3 (Adobe Systems Inc., USA). Levels of *Pgst-4*::GFP were measured as average mean pixel intensity (AMPI) over the whole worm using ImageJ software (Rasband, W.S., U.S. National Institutes of Health, Bethesda, MD, http://rsb.info.nih.gov/ij/). AMPI values were normalized by subtracting the background intensity of the slide as measured by the minimum pixel intensity in each image. Statistical significance was judged using a two-tailed student's t-test in Excel (v.12.2.4, Microsoft, USA).

## Supporting Information

Table S1 Lifespan assays were conducted as described in Methods. Control lifespan varied by experiment, and individual experiments can be identified by control mean lifespan; (+) treatment; (−) DMSO vehicle control. * Mean lifespan of the 90^th^ percentile. ∧ Log-Rank Probability.
(DOC)

Table S2 † Animals were grown from embryos on feeding RNAi vector control bacterial strain L4440 or RNAi bacteria expressing skn-1 dsRNA. Day 0 adults were transferred to treatment plates with 200 µM FUdR to prevent progeny production; (+) treatment; (−) DMSO vehicle control. * Mean lifespan of the 90^th^ percentile. ∧ Log-Rank Probability.
(DOC)

Table S3 CL2166 animals were grown to adulthood and treated for two days as indicated. (+) Treatment; (-) DMSO vehicle control. * AMPI represents the average whole worm background-subtracted mean pixel intensity for the treatment group. † Ratio given is the AMPI for the treatment group relative to the DMSO control. ∧ T-test.
(DOC)

Table S4 # P, plumbagin; O, Oxoline; N, naphthazarin. ∧ (+) Bacteria killed at 65°C for 1 hour; (−) Live bacteria spread onto plates; both (+) and (−) plates contained FUDR to prevent progeny production and control bacterial growth.
(DOC)

## References

[1] Finkel T, Holbrook NJ (2000). Oxidants, oxidative stress and the biology of ageing.. Nature.

[2] Kregel KC, Zhang HJ (2007). An integrated view of oxidative stress in aging: basic mechanisms, functional effects, and pathological considerations.. Am J Physiol Regul Integr Comp Physiol.

[3] Bejarano E, Cuervo AM (2010). Chaperone-mediated autophagy.. Proc Am Thorac Soc.

[4] Head E, Liu J, Hagen TM, Muggenburg BA, Milgram NW (2002). Oxidative damage increases with age in a canine model of human brain aging.. J Neurochem.

[5] Choksi KB, Nuss JE, Boylston WH, Rabek JP, Papaconstantinou J (2007). Age-related increases in oxidatively damaged proteins of mouse kidney mitochondrial electron transport chain complexes.. Free Radic Biol Med.

[6] Ames BN (1989). Endogenous oxidative DNA damage, aging, and cancer.. Free Radic Res Commun.

[7] Bonda DJ, Wang X, Perry G, Nunomura A, Tabaton M (2010). Oxidative stress in Alzheimer disease: a possibility for prevention.. Neuropharmacology.

[8] Munoz MJ, Riddle DL (2003). Positive selection of *Caenorhabditis elegans* mutants with increased stress resistance and longevity.. Genetics.

[9] Ungvari Z, Buffenstein R, Austad SN, Podlutsky A, Kaley G (2008). Oxidative stress in vascular senescence: lessons from successfully aging species.. Front Biosci.

[10] Le Bourg E (2009). Hormesis, aging and longevity.. Biochim Biophys Acta.

[11] Calabrese EJ, Bachmann KA, Bailer AJ, Bolger PM, Borak J (2007). Biological stress response terminology: Integrating the concepts of adaptive response and preconditioning stress within a hormetic dose-response framework.. Toxicol Appl Pharmacol.

[12] McAlister L, Finkelstein DB (1980). Heat shock proteins and thermal resistance in yeast.. Biochem Biophys Res Commun.

[13] Burton V, Mitchell HK, Young P, Petersen NS (1988). Heat shock protection against cold stress of *Drosophila melanogaster*.. Mol Cell Biol.

[14] Lithgow GJ, White TM, Melov S, Johnson TE (1995). Thermotolerance and extended life-span conferred by single-gene mutations and induced by thermal stress.. Proc Natl Acad Sci U S A.

[15] Yashin AI, Cypser JR, Johnson TE, Michalski AI, Boyko SI (2001). Ageing and survival after different doses of heat shock: the results of analysis of data from stress experiments with the nematode worm *Caenorhabditis elegans*.. Mech Ageing Dev.

[16] Cypser JR, Johnson TE (2002). Multiple stressors in Caenorhabditis elegans induce stress hormesis and extended longevity.. J Gerontol A Biol Sci Med Sci.

[17] Plesset J, Palm C, McLaughlin CS (1982). Induction of heat shock proteins and thermotolerance by ethanol in *Saccharomyces cerevisiae*.. Biochem Biophys Res Commun.

[18] Messier AA, Fisher HW (1990). Sensitivity of cultured mammalian cells to oxidative stress: adaptation to repeated exposures of hyperbaric oxygen.. Undersea Biomed Res.

[19] Heidler T, Hartwig K, Daniel H, Wenzel U (2010). *Caenorhabditis elegans* lifespan extension caused by treatment with an orally active ROS-generator is dependent on DAF-16 and SIR-2.1.. Biogerontology.

[20] An JH, Blackwell TK (2003). SKN-1 links *C. elegans* mesendodermal specification to a conserved oxidative stress response.. Genes Dev.

[21] Son TG, Camandola S, Arumugam TV, Cutler RG, Telljohann RS (2010). Plumbagin, a novel Nrf2/ARE activator, protects against cerebral ischemia.. J Neurochem.

[22] Doniach T, Hodgkin J (1984). A sex-determining gene, fem-1, required for both male and hermaphrodite development in *Caenorhabditis elegans*.. Dev Biol.

[23] Keaney M, Matthijssens F, Sharpe M, Vanfleteren J, Gems D (2004). Superoxide dismutase mimetics elevate superoxide dismutase activity *in vivo* but do not retard aging in the nematode *Caenorhabdites elegans*.. Free Radic Biol Med.

[24] Gems D, Riddle DL (2000). Genetic, behavioral and environmental determinants of male longevity in *Caenorhabditis elegans*.. Genetics.

[25] Garigan D, Hsu AL, Fraser AG, Kamath RS, Ahringer J (2002). Genetic analysis of tissue aging in *Caenorhabditis elegans*: a role for heat-shock factor and bacterial proliferation.. Genetics.

[26] Cohen SS, Flaks JG, Barner HD, Loeb MR, Lichtenstein J (1958). The Mode of Action of 5-Fluorouracil and Its Derivatives.. Proc Natl Acad Sci U S A.

[27] Summers WC, Raksin P (1993). A method for selection of mutations at the tdk locus in *Escherichia coli*.. J Bacteriol.

[28] Blum J, Fridovich I (1983). Superoxide, hydrogen peroxide, and oxygen toxicity in two free-living nematode species.. Arch Biochem Biophys.

[29] Leiers B, Kampkotter A, Grevelding CG, Link CD, Johnson TE et al (2003). A stress-responsive glutathione S-transferase confers resistance to oxidative stress in *Caenorhabditis elegans*.. Free Radic Biol Med.

[30] Park S-K, Tedesco PM, Johnson TE (2009). Oxidative stress and longevity in *Caenorhabditis elegans* as mediated by SKN-1.. Aging Cell.

[31] Przybysz AJ, Choe KP, Roberts LJ, Strange K (2009). Increased age reduces DAF-16 and SKN-1 signaling and the hormetic response of *Caenorhabditis elegans* to the xenobiotic juglone.. Mech Ageing Dev.

[32] Wakabayashi N, Slocum SL, Skoko JJ, Shin S, Kensler TW (2010). When NRF2 talks, who's listening?. Antioxid Redox Signal.

[33] Oliveira RP, Porter Abate J, Dilks K, Landis J, Ashraf J (2009). Condition-adapted stress and longevity gene regulation by *Caenorhabditis elegans* SKN-1/Nrf.. Aging Cell.

[34] Honda Y, Honda S (1999). The daf-2 gene network for longevity regulates oxidative stress resistance and Mn-superoxide dismutase gene expression in *Caenorhabditis elegans*.. FASEB J.

[35] Furuyama T, Nakazawa T, Nakano I, Mori N (2000). Identification of the differential distribution patterns of mRNAs and consensus binding sequences for mouse DAF-16 homologues.. Biochem J.

[36] Henderson ST, Johnson TE (2001). daf-16 integrates developmental and environmental inputs to mediate aging in the nematode *Caenorhabditis elegans*.. Curr Biol.

[37] Gilley J, Coffer PJ, Ham J (2003). FOXO transcription factors directly activate bim gene expression and promote apoptosis in sympathetic neurons.. J Cell Biol.

[38] Tullet JMA, Hertweck M, An JH, Baker J, Hwang JY (2008). Direct inhibition of the longevity-promoting factor SKN-1 by insulin-like signaling in *C. elegans*.. Cell.

[39] Murphy CT, McCarroll SA, Bargmann CI, Fraser A, Kamath RS (2003). Genes that act downstream of DAF-16 to influence the lifespan of *Caenorhabditis elegans*.. Nature.

[40] Link CD, Johnson CJ (2002). Reporter transgenes for study of oxidant stress in *Caenorhabditis elegans*.. Methods Enzymol.

[41] Kahn NW, Rea SL, Moyle S, Kell A, Johnson TE (2008). Proteasomal dysfunction activates the transcription factor SKN-1 and produces a selective oxidative-stress response in *Caenorhabditis elegans*.. Biochem J.

[42] Newton LAA, Cowham E, Sharp D, Leslie R, Davis J (2010). Plumbagin: a natural product for smart materials?. New J Chem.

[43] Rattan SI (2008). Principles and practice of hormetic treatment of aging and age-related diseases.. Hum Exp Toxicol.

[44] Calabrese V, Cornelius C, Dinkova-Kostova AT, Calabrese EJ, Mattson MP (2010). Cellular stress responses, the hormesis paradigm, and vitagenes: novel targets for therapeutic intervention in neurodegenerative disorders.. Antioxid Redox Signal.

[45] Balogun E, Hoque M, Gong P, Killeen E, Green CJ (2003). Curcumin activates the haem oxygenase-1 gene via regulation of Nrf2 and the antioxidant-responsive element.. Biochem J.

[46] Garg R, Gupta S, Maru GB (2008). Dietary curcumin modulates transcriptional regulators of phase I and phase II enzymes in benzo[a]pyrene-treated mice: mechanism of its anti-initiating action.. Carcinogenesis.

[47] Dinkova-Kostova AT, Holtzclaw WD, Cole RN, Itoh K, Wakabayashi N (2002). Direct evidence that sulfhydryl groups of Keap1 are the sensors regulating induction of phase 2 enzymes that protect against carcinogens and oxidants.. Proc Natl Acad Sci U S A.

[48] Higgins LG, Kelleher MO, Eggleston IM, Itoh K, Yamamoto M (2009). Transcription factor Nrf2 mediates an adaptive response to sulforaphane that protects fibroblasts in vitro against the cytotoxic effects of electrophiles, peroxides and redox-cycling agents.. Toxicol Appl Pharmacol.

[49] Cypser JR, Johnson TE (2003). Hormesis in *Caenorhabditis elegans* dauer-defective mutants.. Biogerontology.

[50] Castro FA, Mariani D, Panek AD, Eleutherio EC, Pereira MD (2008). Cytotoxicity mechanism of two naphthoquinones (menadione and plumbagin) in *Saccharomyces cerevisiae*.. PLoS One.

[51] Dudas I, Puho E, Czeizel AE (2006). Population-based case-control study of oxoline acid use during pregnancy for birth outcomes.. Congenit Anom (Kyoto).

[52] Zenkova E, Degterev E (2000). 1,2,3,4-Tetrahydro-1,4-dioxo-2,2,3,3-tetrahydroxynaphthalene: A reagent for TLC detection of biogenic amines and a source of ninhydrin reagent.. Pharmaceutical Chemistry Journal.

[53] Brenner S (1974). The genetics of *Caenorhabditis elegans*.. Genetics.

[54] Hosono R (1978). Sterilization and growth inhibition of *Caenorhabditis elegans* by 5-fluorodeoxyuridine.. Exp Gerontol.

[55] Cheadle C, Vawter MP, Freed WJ, Becker KG (2003). Analysis of microarray data using Z score transformation.. J Mol Diagn.

[56] Timmons L, Fire A (1998). Specific interference by ingested dsRNA.. Nature.

