# Supplementary material for: Extension of Lifespan in C. elegans by Naphthoquinones That Act through Stress Hormesis Mechanisms
Source: PLoS One. 2011 Jul 13;6(7):e21922. doi: 10.1371/journal.pone.0021922 (PMC3135594; doi:10.1371/journal.pone.0021922)
Supplement: Table S1 — Lifespan assays were conducted as described in Methods. Control lifespan varied by experiment, and individual experiments can be identified by control mean lifespan; (+) treatment; (−) DMSO vehicle control. * Mean lifespan of the 90th percentile. ∧ Log-Rank Probability. (DOC) [file pone.0021922.s001.doc]

Table S1. C. elegans lifespan on a food source of OP50 in the presence of FUDR.

Plumbagin

| Strain | Dose (µM) |  | Mean lifespan | | |  | | | Maximum lifespan* | | | | | | | | | n | |
| --- | --- | --- | --- | --- | --- | --- | --- | --- | --- | --- | --- | --- | --- | --- | --- | --- | --- | --- | --- |
|  |  |  | + | - | Ratio | | P^ |  | | + | | - | | Ratio | | P^ |  | + | - |
| N2 | 1 | 24.5 | | 24.2 | 1.01 | | 0.7 | | 32.3 | | | 31.2 | | 1.03 | | 0.3 | | 116 | 211 |
|  | 2.5 | 24.8 | | 24.2 | 1.03 | | 0.6 | | 32.8 | | | 31.2 | | 1.05 | | 0.1 | | 105 | 211 |
|  | 5 | 24.8 | | 24.2 | 1.03 | | 0.6 | | 32.1 | | | 31.2 | | 1.03 | | 0.5 | | 112 | 211 |
|  | 7.5 | 24.2 | | 24.2 | 1.00 | | 0.6 | | 31.3 | | | 31.2 | | 1.00 | | 0.9 | | 107 | 211 |
|  | 10 | 22.3 | | 20.5 | 1.09 | | 0.2 | | 28.6 | | | 28.0 | | 1.02 | | 0.2 | | 104 | 69 |
|  |  | 22.0 | | 20.7 | 1.07 | | 0.03 | | 28.6 | | | 27.8 | | 1.03 | | 0.1 | | 98 | 97 |
|  | 25 | 26.5 | | 23.2 | 1.15 | | <0.01 | | 31.1 | | | 29.7 | | 1.05 | | 0.03 | | 137 | 128 |
|  |  | 22.5 | | 20.5 | 1.10 | | 0.08 | | 28.6 | | | 28.0 | | 1.02 | | 0.1 | | 128 | 69 |
|  |  | 22.9 | | 20.7 | 1.11 | | <0.01 | | 29.5 | | | 27.8 | | 1.06 | | <0.01 | | 76 | 97 |
|  |  | 26.9 | | 24.2 | 1.11 | | <0.01 | | 35.3 | | | 31.2 | | 1.13 | | <0.01 | | 118 | 211 |
|  | 50 | 26.0 | | 23.2 | 1.12 | | <0.01 | | 31.3 | | | 29.7 | | 1.05 | | 0.03 | | 122 | 128 |
|  |  | 19.9 | | 20.5 | 0.97 | | 0.02 | | 26.4 | | | 28.0 | | 0.94 | | 0.1 | | 128 | 69 |
|  |  | 23.1 | | 20.7 | 1.12 | | <0.01 | | 31.0 | | | 27.8 | | 1.12 | | <0.01 | | 104 | 97 |
|  | 100 | 17.2 | | 20.5 | 0.84 | | <0.01 | | 23.9 | | | 28.0 | | 0.85 | | <0.01 | | 118 | 69 |
|  |  | 20.8 | | 20.7 | 1.01 | | 0.5 | | 28.4 | | | 27.8 | | 1.02 | | 0.3 | | 101 | 97 |
|  |  | 19.9 | | 22.3 | 0.89 | | <0.01 | | 23.2 | | | 26.5 | | 0.87 | | <0.01 | | 101 | 113 |
|  | 150 | 15.8 | | 20.5 | 0.77 | | <0.01 | |  | |  | |  | |  | | | 127 | 69 |
|  | 200 | 3.3 | | 20.5 | 0.16 | | <0.01 | |  | |  | |  | |  | | | 118 | 69 |
|  | 250 | 2.1 | | 23.2 | 0.09 | | <0.01 | |  | |  | |  | |  | | | 144 | 128 |
|  |  | 2.8 | | 20.5 | 0.14 | | <0.01 | |  | |  | |  | |  | | | 111 | 69 |
|  | 300 | 2.8 | | 20.5 | 0.14 | | <0.01 | |  | |  | |  | |  | | | 93 | 69 |
|  | 400 | 2.0 | | 20.5 | 0.10 | | <0.01 | |  | |  | |  | |  | | | 81 | 69 |
|  | 500 | 3.9 | | 21.5 | 0.18 | | <0.01 | |  | |  | |  | |  | | | 52 | 96 |
|  |  | 2.0 | | 23.2 | 0.09 | | <0.01 | |  | |  | |  | |  | | | 157 | 128 |
| skn-1 (zu135) | 25 | 19.5 | | 18.3 | 1.07 | | 0.1 | | 25.4 | | | 25.0 | | 1.02 | | 0.5 | | 65 | 65 |
|  |  | 19.7 | | 19.6 | 1.01 | | 0.4 | | 28.3 | | | 29.4 | | 0.96 | | 0.3 | | 107 | 99 |
|  |  | 17.0 | | 18.8 | 0.90 | | <0.01 | | 22.5 | | | 25.2 | | 0.89 | | <0.01 | | 112 | 119 |
|  |  | 14.6 | | 17.3 | 0.84 | | <0.01 | | 26.0 | | | 30.0 | | 0.87 | | 0.02 | | 108 | 123 |
| daf-16 (mgDf50); daf-2  (e1370) | 25 | 14.7 | | 14.0 | 1.05 | | 0.4 | | 17.6 | | | 18.4 | | 0.95 | | 0.1 | | 97 | 68 |
|  |  | 15.5 | | 14.2 | 1.09 | | <0.01 | | 19.4 | | | 18.0 | | 1.08 | | <0.01 | | 112 | 80 |
|  |  | 15.8 | | 14.3 | 1.10 | | <0.01 | | 19.0 | | | 17.5 | | 1.09 | | <0.01 | | 111 | 75 |
|  |  | 15.1 | | 14.8 | 1.02 | | 0.97 | | 18.6 | | | 18.8 | | 0.99 | | 0.7 | | 104 | 104 |
| daf-16 (mgDf50) | 10 | 15.3 | | 14.5 | 1.06 | | <0.01 | |  | |  | |  | |  | | | 49 | 73 |
|  | 25 | 14.6 | | 14.5 | 1.01 | | 0.5 | |  | |  | |  | |  | | | 47 | 73 |
|  | 50 | 13.6 | | 14.5 | 0.94 | | <0.01 | |  | |  | |  | |  | | | 48 | 73 |
|  | 100 | 11.2 | | 14.5 | 0.77 | | <0.01 | |  | |  | |  | |  | | | 72 | 73 |

Naphthazarin

| Strain | Dose (µM) |  | Mean lifespan | | | |  | | | | Maximum lifespan* | | | | | | | | | n | | |
| --- | --- | --- | --- | --- | --- | --- | --- | --- | --- | --- | --- | --- | --- | --- | --- | --- | --- | --- | --- | --- | --- | --- |
|  |  |  | + | - | | Ratio | | P^ |  | | | + | - | | Ratio | P^ | |  | | | + | - |
| N2 | 50 | 22.8 | | 22.3 | 1.02 | | | 0.2 | | 26.4 | | | | 26.5 | 1.00 | | 0.8 | | 67 | | | 113 |
|  | 100 | 23.5 | | 21.2 | 1.11 | | | <0.01 | | 32.8 | | | | 29.2 | 1.12 | | <0.01 | | 93 | | | 186 |
|  |  | 24.5 | | 22.3 | 1.10 | | | <0.01 | | 29.6 | | | | 26.5 | 1.12 | | <0.01 | | 98 | | | 113 |
|  |  | 25.4 | | 23.5 | 1.08 | | | <0.01 | | 31.2 | | | | 29.2 | 1.07 | | <0.01 | | 94 | | | 128 |
|  | 200 | 25.3 | | 21.2 | 1.19 | | | <0.01 | | 33.8 | | | | 29.2 | 1.16 | | <0.01 | | 96 | | | 186 |
|  |  | 24.2 | | 22.3 | 1.08 | | | <0.01 | | 30.3 | | | | 26.5 | 1.14 | | <0.01 | | 102 | | | 113 |
|  |  | 25.9 | | 23.5 | 1.10 | | | <0.01 | | 31.4 | | | | 29.2 | 1.08 | | <0.01 | | 97 | | | 128 |
|  | 500 | 23.1 | | 21.2 | 1.09 | | | 0.05 | | 30.4 | | | | 29.2 | 1.04 | | 0.1 | | 94 | | | 186 |
|  |  | 27.3 | | 22.3 | 1.22 | | | <0.01 | | 33.1 | | | | 26.5 | 1.25 | | <0.01 | | 111 | | | 113 |
|  |  | 26.8 | | 23.5 | 1.14 | | | <0.01 | | 31.6 | | | | 29.2 | 1.08 | | <0.01 | | 66 | | | 128 |
| skn-1 (zu135) | 200 | 10.9 | | 18.3 | 0.59 | | | <0.01 | | 14.3 | | | | 25.0 | 0.57 | | <0.01 | | 64 | | | 65 |
|  |  | 13.3 | | 19.6 | 0.68 | | | <0.01 | | 16.0 | | | | 29.4 | 0.54 | | <0.01 | | 103 | | | 99 |
|  |  | 8.5 | | 18.8 | 0.45 | | | <0.01 | | 11.3 | | | | 25.2 | 0.45 | | <0.01 | | 106 | | | 119 |
|  |  | 8.4 | | 17.3 | 0.49 | | | <0.01 | | 11.6 | | | | 30.0 | 0.39 | | <0.01 | | 110 | | | 123 |
| daf-16 (mgDf50); daf-2  (e1370) | 200 | 15.7 | | 14.0 | 1.12 | | | <0.01 | | 18.6 | | | | 18.4 | 1.01 | | 0.7 | | 107 | | | 68 |
|  |  | 15.9 | | 14.2 | 1.12 | | | <0.01 | | 19.0 | | | | 18.0 | 1.06 | | <0.01 | | 116 | | | 80 |
|  |  | 16.1 | | 14.3 | 1.12 | | | <0.01 | | 19.0 | | | | 17.5 | 1.09 | | <0.01 | | 105 | | | 75 |
|  |  | 15.9 | | 14.8 | 1.07 | | | 0.01 | | 19.5 | | | | 18.8 | 1.04 | | 0.3 | | 103 | | | 104 |

Oxoline

| Strain | Dose (µM) |  | Mean lifespan | | | |  | | | | Maximum lifespan* | | | | | | | | | n | | |
| --- | --- | --- | --- | --- | --- | --- | --- | --- | --- | --- | --- | --- | --- | --- | --- | --- | --- | --- | --- | --- | --- | --- |
|  |  |  | + | - | | Ratio | | P^ |  | | | + | - | | Ratio | P^ | |  | | | + | - |
| N2 | 50 | 24.7 | | 24.2 | 1.02 | | | 0.9 | | 31.5 | | | | 31.2 | 1.01 | | 0.9 | | 103 | | | 211 |
|  | 100 | 21.6 | | 21.1 | 1.02 | | | 0.6 | | 28.0 | | | | 30.4 | 0.92 | | 0.05 | | 61 | | | 59 |
|  |  | 20.8 | | 19.1 | 1.09 | | | 0.02 | | 30.6 | | | | 26.6 | 1.15 | | <0.01 | | 93 | | | 108 |
|  |  | 24.7 | | 24.2 | 1.02 | | | 0.6 | | 32.7 | | | | 31.2 | 1.05 | | 0.1 | | 108 | | | 211 |
|  | 500 | 24.7 | | 21.5 | 1.15 | | | <0.01 | | 32.8 | | | | 29.7 | 1.10 | | <0.01 | | 95 | | | 96 |
|  |  | 21.9 | | 19.1 | 1.15 | | | <0.01 | | 29.8 | | | | 26.6 | 1.12 | | <0.01 | | 98 | | | 108 |
|  |  | 27.0 | | 26.1 | 1.03 | | | <0.01 | | 33.4 | | | | 29.7 | 1.12 | | <0.01 | | 102 | | | 108 |
|  | 1000 | 27.3 | | 24.2 | 1.13 | | | <0.01 | | 36.8 | | | | 31.2 | 1.18 | | <0.01 | | 101 | | | 211 |
|  |  | 28.6 | | 26.1 | 1.10 | | | <0.01 | | 37.0 | | | | 29.7 | 1.24 | | <0.01 | | 94 | | | 108 |
|  |  | 21.9 | | 19.5 | 1.12 | | | <0.01 | | 28.9 | | | | 23.3 | 1.24 | | <0.01 | | 71 | | | 61 |
| skn-1 (zu135) | 500 | 19.7 | | 18.3 | 1.08 | | | 0.03 | | 26.6 | | | | 25.0 | 1.06 | | 0.2 | | 46 | | | 65 |
|  |  | 20.3 | | 19.6 | 1.04 | | | 0.2 | | 30.1 | | | | 29.4 | 1.02 | | 0.5 | | 101 | | | 99 |
|  |  | 20.8 | | 18.8 | 1.11 | | | <0.01 | | 30.2 | | | | 25.2 | 1.20 | | <0.01 | | 109 | | | 119 |
|  |  | 21.4 | | 17.3 | 1.23 | | | <0.01 | | 32.5 | | | | 30.0 | 1.08 | | <0.01 | | 117 | | | 123 |
| daf-16 (mgDf50); daf-2  (e1370) | 500 | 15.3 | | 14.0 | 1.09 | | | 0.01 | | 18.8 | | | | 18.4 | 1.02 | | 0.6 | | 74 | | | 68 |
|  |  | 15.9 | | 14.2 | 1.12 | | | <0.01 | | 19.2 | | | | 18.0 | 1.07 | | <0.01 | | 121 | | | 80 |
|  |  | 15.7 | | 14.3 | 1.10 | | | <0.01 | | 19.2 | | | | 17.5 | 1.10 | | <0.01 | | 103 | | | 75 |
|  |  | 16.6 | | 14.8 | 1.12 | | | <0.01 | | 21.0 | | | | 18.8 | 1.12 | | <0.01 | | 79 | | | 104 |

Menadione

| Strain | Dose (µM) | Mean lifespan | | | |  | n | |
| --- | --- | --- | --- | --- | --- | --- | --- | --- |
|  |  | + | - | Ratio | P^ |  | + | - |
| N2 | 25 | 21.8 | 21.9 | 0.99 | 0.1 |  | 125 | 133 |
|  | 50 | 24.2 | 23.2 | 1.05 | 0.3 |  | 137 | 128 |
|  |  | 19.8 | 21.9 | 0.90 | <0.01 |  | 115 | 133 |
|  | 100 | 19.3 | 25.1 | 0.77 | <0.01 |  | 59 | 61 |
|  |  | 20.6 | 23.2 | 0.89 | <0.01 |  | 115 | 128 |
|  |  | 17.8 | 21.9 | 0.81 | <0.01 |  | 119 | 133 |
|  | 150 | 18.4 | 21.9 | 0.84 | <0.01 |  | 114 | 133 |
|  | 200 | 16.4 | 21.9 | 0.75 | <0.01 |  | 108 | 133 |
|  | 250 | 15.0 | 21.9 | 0.69 | <0.01 |  | 108 | 133 |
|  | 300 | 15.0 | 21.9 | 0.68 | <0.01 |  | 109 | 133 |
|  | 400 | 12.9 | 21.9 | 0.59 | <0.01 |  | 76 | 133 |
|  | 500 | 13.1 | 21.5 | 0.61 | <0.01 |  | 82 | 96 |
|  |  | 14.0 | 21.9 | 0.64 | <0.01 |  | 76 | 133 |

5-Hydroxy-1-tetralone

| Strain | Dose (µM) | Mean lifespan | | | |  | n | |
| --- | --- | --- | --- | --- | --- | --- | --- | --- |
|  |  | + | - | Ratio | P^ |  | + | - |
| N2 | 100 | 20.4 | 21.1 | 0.96 | <0.01 |  | 74 | 59 |
|  | 500 | 21.9 | 21.5 | 1.02 | 0.4 |  | 92 | 96 |

7-Hydroxy-1-tetralone

| Strain | Dose (µM) | Mean lifespan | | | |  | n | |
| --- | --- | --- | --- | --- | --- | --- | --- | --- |
|  |  | + | - | Ratio | P^ |  | + | - |
| N2 | 100 | 18.5 | 21.1 | 0.87 | <0.01 |  | 77 | 59 |
|  | 500 | 20.0 | 21.5 | 0.93 | <0.01 |  | 108 | 96 |

5,8-Dimethyoxy-1-tetralone

| Strain | Dose (µM) | Mean lifespan | | | |  | n | |
| --- | --- | --- | --- | --- | --- | --- | --- | --- |
|  |  | + | - | Ratio | P^ |  | + | - |
| N2 | 60 | 25.2 | 24.6 | 1.02 | 0.6 |  | 55 | 79 |
|  | 100 | 20.6 | 20.8 | 0.99 | 0.6 |  | 40 | 71 |
|  |  | 22.3 | 21.1 | 1.06 | 0.8 |  | 73 | 59 |
|  | 200 | 24.1 | 25.7 | 0.94 | 0.1 |  | 47 | 36 |
|  |  | 21.9 | 21.2 | 1.03 | 0.9 |  | 91 | 186 |
|  | 500 | 22.4 | 21.2 | 1.06 | 0.2 |  | 93 | 186 |
