# Supplementary material for: Extension of Lifespan in C. elegans by Naphthoquinones That Act through Stress Hormesis Mechanisms
Source: PLoS One. 2011 Jul 13;6(7):e21922. doi: 10.1371/journal.pone.0021922 (PMC3135594; doi:10.1371/journal.pone.0021922)
Supplement: Table S2 — † Animals were grown from embryos on feeding RNAi vector control bacterial strain L4440 or RNAi bacteria expressing skn-1 dsRNA. Day 0 adults were transferred to treatment plates with 200 µM FUdR to prevent progeny production; (+) treatment; (−) DMSO vehicle control. * Mean lifespan of the 90th percentile. ∧ Log-Rank Probability. (DOC) [file pone.0021922.s002.doc]

Table S2. Lifespan data for N2 hermaphrodites under indicated RNAi conditions.

Plumbagin

| Dose (µM) | RNAi | Mean lifespan (days) | | | |  | Maximum lifespan* (days) | | | |  | n | |
| --- | --- | --- | --- | --- | --- | --- | --- | --- | --- | --- | --- | --- | --- |
|  |  | + | - | Ratio | P^ |  | + | - | Ratio | P^ |  | + | - |
| 10 | L4440 | 19.8 | 17.7 | 1.12 | <0.01 |  | 26.1 | 23.0 | 1.14 | <0.01 |  | 146 | 189 |
|  | skn-1 | 16.0 | 16.5 | 0.97 | 0.07 |  | 17.9 | 18.6 | 0.96 | 0.1 |  | 134 | 224 |
| 25 | L4440 | 21.1 | 17.7 | 1.19 | <0.01 |  | 27.4 | 23.0 | 1.19 | <0.01 |  | 109 | 189 |
|  | skn-1 | 16.3 | 16.5 | 0.99 | 0.1 |  | 18.3 | 18.6 | 0.98 | 0.4 |  | 117 | 224 |
|  | L4440 | 21.8 | 19.8 | 1.10 | <0.01 |  | 28.5 | 24.9 | 1.14 | <0.01 |  | 127 | 80 |
|  | skn-1 | 18.1 | 17.8 | 1.02 | 0.3 |  | 22.2 | 22.2 | 1.00 | 1.0 |  | 122 | 125 |
|  | L4440 | 19.8 | 17.5 | 1.13 | <0.01 |  | 27.4 | 24.6 | 1.12 | 0.02 |  | 67 | 86 |
|  | skn-1 | 14.9 | 13.8 | 1.08 | <0.01 |  | 19.2 | 17.6 | 1.09 | 0.1 |  | 110 | 82 |
| 50 | L4440 | 20.9 | 17.8 | 1.18 | <0.01 |  | 27.9 | 23.0 | 1.21 | <0.01 |  | 96 | 189 |
|  | skn-1 | 11.8 | 16.5 | 0.72 | <0.01 |  | 18.0 | 18.6 | 0.97 | 0.4 |  | 84 | 224 |
| 100 | L4440 | 16.1 | 17.7 | 0.91 | 0.3 |  | 23.0 | 23.0 | 1.00 | 0.9 |  | 118 | 189 |
|  | skn-1 | 6.6 | 16.5 | 0.40 | <0.01 |  | 11.9 | 18.6 | 0.64 | <0.01 |  | 108 | 224 |
|  | L4440 | 19.7 | 21.0 | 0.94 | 0.1 |  | 27.8 | 29.6 | 0.94 | 0.03 |  | 101 | 109 |
|  | skn-1 | 11.8 | 17.2 | 0.68 | <0.01 |  | 17.6 | 22.6 | 0.78 | <0.01 |  | 111 | 104 |
| 200 | L4440 | 7.3 | 17.7 | 0.41 | <0.01 |  | 16.9 | 23.0 | 0.74 | <0.01 |  | 116 | 189 |
|  | skn-1 | 3.4 | 16.5 | 0.21 | <0.01 |  | 5.0 | 18.6 | 0.27 | <0.01 |  | 120 | 224 |
| 300 | L4440 | 3.9 | 17.7 | 0.22 | <0.01 |  | 5.2 | 23.0 | 0.23 | <0.01 |  | 112 | 189 |
|  | skn-1 | 3.0 | 16.5 | 0.18 | <0.01 |  | 3.0 | 18.6 | 0.16 | <0.01 |  | 115 | 224 |

Naphthazarin

| Dose (µM) | RNAi | Mean lifespan (days) | | | |  | Maximum lifespan* (days) | | | |  | n | |
| --- | --- | --- | --- | --- | --- | --- | --- | --- | --- | --- | --- | --- | --- |
|  |  | + | - | Ratio | P^ |  | + | - | Ratio | P^ |  | + | - |
| 200 | L4440 | 25.5 | 21.0 | 1.22 | <0.01 |  | 33.2 | 29.5 | 1.12 | <0.01 |  | 98 | 109 |
|  | skn-1 | 16.3 | 17.2 | 0.94 | <0.01 |  | 19.8 | 22.6 | 0.88 | <0.01 |  | 108 | 104 |
|  | L4440 | 23.0 | 19.8 | 1.16 | <0.01 |  | 27.1 | 24.9 | 1.09 | 0.0159 |  | 88 | 80 |
|  | skn-1 | 11.8 | 17.8 | 0.66 | <0.01 |  | 16.0 | 22.2 | 0.72 | <0.01 |  | 127 | 125 |
|  | L4440 | 23.9 | 17.5 | 1.37 | <0.01 |  | 30.9 | 24.6 | 1.26 | <0.01 |  | 80 | 86 |
|  | skn-1 | 15.0 | 13.8 | 1.08 | <0.01 |  | 18.8 | 17.6 | 1.07 | 0.3998 |  | 113 | 82 |
| 500 | L4440 | 21.6 | 19.8 | 1.09 | <0.01 |  | 24.9 | 24.9 | 1.00 | 0.8024 |  | 133 | 80 |
|  | skn-1 | 4.1 | 17.8 | 0.23 | <0.01 |  | 4.8 | 22.2 | 0.22 | <0.01 |  | 95 | 125 |

Oxoline

| Dose (µM) | RNAi | Mean lifespan (days) | | | |  | Maximum lifespan* (days) | | | |  | n | |
| --- | --- | --- | --- | --- | --- | --- | --- | --- | --- | --- | --- | --- | --- |
|  |  | + | - | Ratio | P^ |  | + | - | Ratio | P^ |  | + | - |
| 500 | L4440 | 25.9 | 17.7 | 1.46 | <0.01 |  | 33.5 | 23.0 | 1.46 | <0.01 |  | 117 | 189 |
|  | skn-1 | 17.7 | 16.5 | 1.07 | <0.01 |  | 20.9 | 18.6 | 1.12 | <0.01 |  | 105 | 224 |
|  | L4440 | 24.9 | 19.8 | 1.25 | <0.01 |  | 30.3 | 24.9 | 1.22 | <0.01 |  | 109 | 80 |
|  | skn-1 | 23.7 | 17.8 | 1.33 | <0.01 |  | 29.0 | 22.2 | 1.31 | <0.01 |  | 40 | 125 |
|  | L4440 | 25.3 | 20.3 | 1.24 | <0.01 |  | 32.5 | 20.3 | 1.60 | <0.01 |  | 106 | 117 |
|  | skn-1 | 18.0 | 15.2 | 1.18 | <0.01 |  | 20.2 | 18.2 | 1.11 | <0.01 |  | 124 | 131 |
| 1000 | L4440 | 25.3 | 17.7 | 1.43 | <0.01 |  | 32.3 | 23.0 | 1.41 | <0.01 |  | 115 | 189 |
|  | skn-1 | 25.6 | 16.5 | 1.56 | <0.01 |  | 31.9 | 18.6 | 1.71 | <0.01 |  | 113 | 224 |
