# Supplementary material for: Extension of Lifespan in C. elegans by Naphthoquinones That Act through Stress Hormesis Mechanisms
Source: PLoS One. 2011 Jul 13;6(7):e21922. doi: 10.1371/journal.pone.0021922 (PMC3135594; doi:10.1371/journal.pone.0021922)
Supplement: Table S3 — CL2166 animals were grown to adulthood and treated for two days as indicated. (+) Treatment; (-) DMSO vehicle control. * AMPI represents the average whole worm background-subtracted mean pixel intensity for the treatment group. † Ratio given is the AMPI for the treatment group relative to the DMSO control. ∧ T-test. (DOC) [file pone.0021922.s003.doc]

Table S3. Levels of pgst-4:GFP in CL2166 hermaphrodites measured after two days on indicated treatments.

Plumbagin

| Dose (µM) | AMPI + Std dev * | | Ratio^†^ | P ^ | n | |
| --- | --- | --- | --- | --- | --- | --- |
|  | + | - |  |  | + | - |
| 1 | 12.8±2.9 | 13.6±3.1 | 0.94 | 0.3 | 29 | 28 |
| 5 | 13.1±2.9 | 13.6±3.1 | 0.97 | 0.6 | 27 | 28 |
| 10 | 14.9±3.0 | 13.6±3.1 | 1.10 | 0.09 | 31 | 28 |
|  | 13.5±2.7 | 13.2±3.4 | 1.02 | 0.7 | 32 | 35 |
| 15 | 16.7±3.4 | 13.6±3.1 | 1.23 | 0.001 | 25 | 28 |
| 20 | 17.6±3.4 | 13.6±3.1 | 1.29 | <0.01 | 31 | 28 |
| 25 | 15.2±3.0 | 9.2±1.9 | 1.66 | <0.01 | 33 | 28 |
|  | 19.4±4.8 | 13.6±3.1 | 1.43 | <0.01 | 26 | 28 |
|  | 21.5±6.9 | 13.2±3.4 | 1.63 | <0.01 | 34 | 35 |
| 35 | 24.2±6.0 | 13.6±3.1 | 1.79 | <0.01 | 30 | 28 |
| 50 | 31.5±13.4 | 11.5±2.3 | 2.73 | <0.01 | 30 | 27 |
|  | 31.2±7.0 | 9.2±1.9 | 3.41 | <0.01 | 33 | 28 |
|  | 26.6±8.8 | 13.6±3.1 | 1.96 | <0.01 | 29 | 28 |
| 100 | 26.2±5.6 | 5.3±1.2 | 4.98 | <0.01 | 24 | 25 |
|  | 25.8±6.9 | 5.5±1.0 | 4.69 | <0.01 | 21 | 29 |
|  | 36.8±7.0 | 13.1±1.9 | 2.82 | <0.01 | 27 | 36 |
|  | 21.7±5.9 | 5.2±0.8 | 4.17 | <0.01 | 22 | 25 |
|  | 38.3±8.0 | 11.5±2.3 | 3.32 | <0.01 | 26 | 27 |
| 150 | 37.9±11.4 | 9.2±1.9 | 4.14 | <0.01 | 23 | 28 |
|  | 39.5±18.9 | 13.2±3.4 | 2.99 | <0.01 | 29 | 35 |
|  | 32.2±13.1 | 6.8±1.0 | 4.77 | <0.01 | 17 | 32 |

Naphthazarin

| Dose (µM) | AMPI + Std dev * | | Ratio^†^ | P ^ | n | |
| --- | --- | --- | --- | --- | --- | --- |
|  | + | - |  |  | + | - |
| 50 | 12.9±2.2 | 12.8±2.5 | 1.01 | 0.8 | 33 | 40 |
| 100 | 12.8±2.0 | 8.2±1.6 | 1.57 | <0.01 | 30 | 30 |
|  | 11.2±1.7 | 8.7±1.1 | 1.28 | <0.01 | 35 | 36 |
| 200 | 13.3±1.6 | 8.2±1.6 | 1.63 | <0.01 | 30 | 30 |
| 500 | 16.0±2.7 | 8.2±1.6 | 1.96 | <0.01 | 30 | 30 |
|  | 11.3±1.7 | 8.7±1.1 | 1.29 | <0.01 | 35 | 36 |

Oxoline

| Dose (µM) | AMPI + Std dev * | | Ratio^†^ | P ^ | n | |
| --- | --- | --- | --- | --- | --- | --- |
|  | + | - |  |  | + | - |
| 100 | 6.0±1.1 | 5.3±1.2 | 1.13 | 0.04 | 22 | 25 |
|  | 5.8±0.8 | 5.2±0.8 | 1.12 | <0.01 | 24 | 25 |
|  | 11.5±2.4 | 9.2±1.9 | 1.25 | <0.01 | 16 | 28 |
| 500 | 9.9±2.0 | 5.5±1.0 | 1.80 | <0.01 | 22 | 29 |
|  | 16.1±3.0 | 13.1±1.9 | 1.23 | <0.01 | 36 | 36 |
|  | 14.4±3.0 | 9.2±1.9 | 1.58 | <0.01 | 33 | 28 |
| 1000 | 9.2±2.9 | 5.5±1.0 | 1.67 | <0.01 | 20 | 29 |
|  | 16.6±3.5 | 6.8±1.0 | 2.45 | <0.01 | 41 | 32 |
|  | 15.5±4.0 | 7.4±1.6 | 2.09 | <0.01 | 39 | 45 |

Menadione

| Dose (µM) | AMPI + Std dev * | | Ratio^†^ | P ^ | n | |
| --- | --- | --- | --- | --- | --- | --- |
|  | + | - |  |  | + | - |
| 50 | 15.2±2.3 | 13.7±2.1 | 1.11 | 0.03 | 18 | 32 |
| 100 | 8.9±1.5 | 5.2±0.8 | 1.70 | <0.01 | 20 | 25 |
|  | 21.3±4.8 | 13.2±3.4 | 1.61 | <0.01 | 40 | 35 |
|  | 15.0±3.1 | 7.4±1.6 | 2.02 | <0.01 | 40 | 45 |
| 250 | 24.5±5.0 | 13.2±3.4 | 1.86 | <0.01 | 40 | 35 |
|  | 11.7±1.9 | 5.3±0.6 | 2.20 | <0.01 | 24 | 21 |
|  | 18.3±4.1 | 6.8±1.0 | 2.72 | <0.01 | 37 | 32 |

5-Hydroxy-1-tetralone

| Dose (µM) | AMPI + Std dev * | | Ratio^†^ | P ^ | n | |
| --- | --- | --- | --- | --- | --- | --- |
|  | + | - |  |  | + | - |
| 100 | 6.0±1.4 | 5.3±1.2 | 1.14 | 0.1 | 12 | 25 |
|  | 7.1±1.1 | 7.0±1.1 | 1.01 | 0.9 | 32 | 35 |
| 500 | 14.0±2.1 | 13.1±1.9 | 1.07 | 0.1 | 14 | 36 |
|  | 8.3±2.0 | 7.0±1.1 | 1.19 | <0.01 | 30 | 35 |

7-Hydroxy-1-tetralone

| Dose (µM) | AMPI + Std dev * | | Ratio^†^ | P ^ | n | |
| --- | --- | --- | --- | --- | --- | --- |
|  | + | - |  |  | + | - |
| 100 | 6.3±1.5 | 5.3±1.2 | 1.19 | 0.02 | 21 | 25 |
|  | 7.2±0.8 | 7.0±1.1 | 1.02 | 0.5 | 32 | 35 |
| 500 | 14.3±2.1 | 13.1±1.9 | 1.10 | 0.02 | 23 | 36 |
|  | 8.2±1.8 | 7.0±1.1 | 1.17 | <0.01 | 32 | 35 |

5,8-Dimethoxy-1-tetralone

| Dose (µM) | AMPI + Std dev * | | Ratio^†^ | P ^ | n | |
| --- | --- | --- | --- | --- | --- | --- |
|  | + | - |  |  | + | - |
| 100 | 5.7±0.7 | 5.2±0.8 | 1.09 | 0.03 | 23 | 25 |
|  | 7.2±2.7 | 7.0±1.1 | 1.03 | 0.7 | 28 | 35 |
| 500 | 13.2±2.3 | 13.1±1.9 | 1.01 | 0.8 | 28 | 36 |
|  | 11.2±2.2 | 9.2±1.9 | 1.22 | <0.01 | 16 | 28 |
|  | 7.5±1.2 | 7.0±1.1 | 1.07 | 0.1 | 32 | 35 |
