# Supplementary material for: Extension of Lifespan in C. elegans by Naphthoquinones That Act through Stress Hormesis Mechanisms
Source: PLoS One. 2011 Jul 13;6(7):e21922. doi: 10.1371/journal.pone.0021922 (PMC3135594; doi:10.1371/journal.pone.0021922)
Supplement: Table S4 — # P, plumbagin; O, Oxoline; N, naphthazarin. ∧ (+) Bacteria killed at 65°C for 1 hour; (−) Live bacteria spread onto plates; both (+) and (−) plates contained FUDR to prevent progeny production and control bacterial growth. (DOC) [file pone.0021922.s004.doc]

Table S4. Effects of plumbagin, oxoline and naphthalene on lifespan of N2 hermaphrodites fed heat-killed bacteria.

| Experiment | Treatment^#^ | Heat-killed bacteria^ | Mean lifespan (days) | | % control lifespan | P  (Log-Rank) | n (censored) |
| --- | --- | --- | --- | --- | --- | --- | --- |
|  |  |  | Treated | Control |  |  |  |
| #1 | DMSO | - | 20.65 |  |  |  | 97 (2) |
|  |  | + | 27.77 | 20.65 | 134% | <0.001 | 84 |
|  | P 10 µM | - | 22.01 | 20.65 | 107% | 0.03 | 98 |
|  |  | + | 26.57 | 27.77 | 96% | 0.05 | 101 |
|  | P 25 µM | - | 22.89 | 20.65 | 111% | <0.001 | 76 (1) |
|  |  | + | 24.02 | 27.77 | 86% | <0.001 | 92 (5) |
|  | P 50 µM | - | 23.14 | 20.65 | 112% | <0.001 | 104 |
|  |  | + | 18.68 | 27.77 | 67% | <0.001 | 104 |
|  | P 100 µM | - | 20.84 | 20.65 | 101% | 0.5 | 101 (1) |
|  |  | + | 10.03 | 27.77 | 36% | <0.001 | 99 (2) |
| #2 | DMSO (1) | - | 24.76 |  |  |  | 106 (1) |
|  |  | + | 26.14 | 24.76 | 106% | 0.02 | 122 |
|  | P 1 µM | - | 24.48 | 24.16 | 101% | 0.8 | 116 |
|  |  | + | 25.47 | 26.01 | 98% | 0.3 | 116 |
|  | P 2.5 µM | - | 24.79 | 24.16 | 103% | 0.6 | 105 |
|  |  | + | 24.65 | 26.01 | 95% | 0.02 | 71 (2) |
|  | P 5 µM | - | 24.79 | 24.16 | 103% | 0.6 | 112 |
|  |  | + | 24.55 | 26.01 | 94% | 0.008 | 110 |
|  | P 7.5 µM | - | 24.18 | 24.16 | 100% | 0.6 | 107 (1) |
|  |  | + | 23.95 | 26.01 | 92% | <0.001 | 114 |
|  | P 25 µM | - | 26.93 | 24.16 | 111% | <0.001 | 118 |
|  |  | + | 24.76 | 26.01 | 95% | 0.09 | 118 (2) |
|  | O 50 µM | - | 24.71 | 24.16 | 102% | 0.9 | 103 |
|  |  | + | 23.24 | 26.01 | 89% | <0.001 | 104 (1) |
|  | O 100 µM | - | 24.72 | 24.16 | 102% | 0.6 | 108 |
|  |  | + | 25.33 | 26.01 | 97% | 0.2 | 102 (1) |
|  | O 500 µM | - | 27.32 | 24.16 | 113% | <0.001 | 101 |
|  |  | + | 27.25 | 26.01 | 105% | 0.01 | 107 (1) |
|  | DMSO (2) | - | 23.56 |  |  |  | 105 |
|  |  | + | 25.87 | 23.56 | 110% | 0.007 | 84 (24) |
| #3 | DMSO | - | 22.29 | - | - | - | 113 (1) |
|  |  | + | 30.86 | 22.29 | 138% | <0.001 | 83 |
|  | P 100 µM | - | 19.94 | 22.29 | 89% | <0.001 | 101 (1) |
|  |  | + | 21.51 | 30.86 | 70% | <0.001 | 87 (6) |
|  | N 50 µM | - | 22.82 | 22.29 | 102% | 0.2 | 67 |
|  |  | + | 30.35 | 30.86 | 98% | 0.5 | 86 |
|  | N 100 µM | - | 24.5 | 22.29 | 110% | <0.001 | 98 (6) |
|  |  | + | 21.35 | 30.86 | 69% | <0.001 | 96 |
|  | N 200 µM | - | 24.17 | 22.29 | 108% | <0.001 | 102 (6) |
|  |  | + | 14.47 | 30.86 | 47% | <0.001 | 88 |
|  | N 500 µM | - | 27.3 | 22.29 | 122% | <0.001 | 111 |
|  |  | + | 8.8 | 30.86 | 29% | <0.001 | 75 |
